# Advantageous environment of micro-patterned, high-density complementary metal–oxide–semiconductor electrode array for spiral ganglion neurons cultured *in vitro*

**DOI:** 10.1038/s41598-018-25814-w

**Published:** 2018-05-10

**Authors:** Viktorija Radotić, Dries Braeken, Petar Drviš, Marta Mattotti, Damir Kovačić

**Affiliations:** 1 Laboratory for Biophysics and Medical Neuroelectronics, Department of Physics, University of Split, Faculty of Science, R.Boškovića 33, HR-21000 Split, Croatia; 2 The Center of Research Excellence for Science and Technology Integrating Mediterranean region (STIM), University of Split, Poljička 35, HR-21000 Split, Croatia; 3 Cell and Tissue Technologies group, Life Science Technologies department, Imec, Kapeldreef 75, B-3001 Leuven, Belgium; 4 University Hospital Centre Split, Department of Otorhinolaryngology & Head and Neck Surgery, Spinčićeva 1, HR-21000 Split, Croatia; 5 Speech and Hearing Research Laboratory, University of Split, School of Medicine, Šoltanska 2, HR-21000 Split, Croatia

## Abstract

This study investigated micro-patterned, high-density complementary metal–oxide–semiconductor (CMOS) electrode array to be used as biologically permissive environment for organization, guidance and electrical stimulation of spiral ganglion neurons (SGN). SGNs extracted and isolated from cochleae of P5-P7 rat pups and adult guinea pigs were cultured 1, 4 and 7 days *in vitro* on glass coverslips (control) and CMOS electrode array. The cultures were analyzed visually and immunohistochemically for SGN presence, outgrowth, neurite alignment, neurite length, neurite asymmetry as well as the contact of a neuronal soma and neurites with the micro-electrodes. Our findings indicate that topographical environment of CMOS chip with micro-patterned pillars enhanced growth, survival, morphology, neural orientation and alignment of SGNs *in vitro* compared to control. Smaller spacing (0.8–1.6 µm) between protruding pillars on CMOS led SGNs to develop structured and guided neurites oriented along three topographical axes separated by 60°. We found morphological basis for positioning of the micro-electrodes on the chip that was appropriate for direct contact of SGNs with them. This configuration allowed CMOS electrode array to electrically stimulate the SGN whose responses were observed with live Fluo 4 calcium imaging.

## Introduction

The cochlear implant (CI) is currently the prevailing neuro-prosthetic treatment for partial restoration of hearing in deaf people. Despite its significant success, it is rather limited in terms of full recovery of sensorineural hearing loss (SNHL), caused by the loss or impairment of either hair cells, i.e. sensory cells in the inner ear or spiral ganglion neurons (SGN), or both, which affects majority of hearing impaired patients^1–3^. A CI comprises a linear electrode array containing up to 26 electrodes that is surgically inserted into the cochlea, typically within the perilymph-filled scala tympani^4^. This arrangement functionally bypasses some or all of approximately 3,400 malfunctioned hair cells by directly stimulating preserved spiral ganglion neurons which forms the auditory nerve^5,6^. Due to the limited spectro-temporal information delivered with electrical stimulation, speech perception as well as listening to music are still rather inadequate in the majority of CI users, especially in real-world settings^7,8^. The neuron-electrode interface plays a crucial role in neural coding of the auditory information. Our approach to overcome major constraint of non-focal and non-selective electrical stimulation of SGNs, located in the auditory nerve, is based on substantial increase in the number of stimulating electrodes. The significant reduction in the size of stimulating electrodes allows comparability to the cellular size of SGNs. This approach targets not only decreased SGN survival which largely depends on the duration and etiology of deafness, but also the anatomical gap between the implanted electrode array and the stimulated regions of the auditory nerve^3,5–7,9–11^. Survival, regeneration and guidance of spiral ganglion neurons were the focus of recent studies^10,12–16^. However, controlled guidance and patterned growth of the auditory neurons to form intimate contact with stimulating electrodes remain elusive. Several studies reported chemical cues, like nerve growth factors or neurotrophins for regeneration and guidance of SGN^10,17–19^. Due to safety issues and the degradation of the chemicals such an approach may not be desirable for clinical applications. For this reason, several *in vitro* studies demonstrated topographical cues for organization and guidance of neurites^18,20–29^. One of them, the so called the “passive model”- silicon micro-pillar substrates (MPS), is composed of biocompatible material with surfaces proven to control neuronal growth and morphology *in vitro*^23,25,27,28,30^. Studies by Mattotti *et al*.^27^ with SGNs isolated from neonatal rats and Repić *et al*.^29^ with neonatal and adult dorsal root ganglion neurons demonstrated the capacity of micro-pillar substrates to support normal neuronal growth, enhance neurite alignment, promote outgrowth and induce specific morphologies on micro-pillars. Furthermore, several recent attempts to embed microelectrodes into multi-electrode arrays (MEA) using complementary metal–oxide–semiconductor (CMOS) technology yield promising results^31–35^. MPS can attract and guide spiral ganglion neurons but cannot ensure long-term neuronal survival due to absence of electrical stimulation. *In vivo* studies have demonstrated that electrical stimulation from CI electrodes promotes survival of SGN^36,37^. Another important issue, specifically investigated in this study, is whether neuronal morphology is affected by the position of CMOS micro-electrodes’ embedded by CMOS among the pillars. In contrast, MPS is a “passive substrate” containing only pillars and cannot provide information on the effect of the position of micro-electrodes’. Thus, a step further from MPSs is an “active model” which is a substrate incorporating CMOS with a potential to simultaneously stimulate neurons and record their electrical activity. In this study, we developed and used CMOS chip substrates consisting of protruding pillars and pillar-like titanium nitride (TiN) micro-electrodes between 1.4 and 4.8 µm in diameter and with a spacing between 0.8 and 1.6 µm with a height of 1 µm. These dimensions are similar to typical mammalian cell bodies^31–33^. Furthermore, these micro-electrodes incorporate *in situ* circuits for voltage and current stimulation with an amplification transistor, all embedded in a silicon oxide environment.

For successful electrical stimulation it is crucial to investigate electrophysiologically relevant morphological specializations of SGN cultured *in vitro* in this active model and to assess whether its topography can serve as favorable environment for the organization and guidance of SGNs. Adult-derived SGNs from guinea pigs were cultured on these CMOS substrates and were compared with SGN cultures from neonatal (P5-P7) rat pups in order to assess the effect of micro-patterned surfaces of CMOS chips on neuronal presence, survival, morphology and alignment as well as the effect of age on sensitivity to topography. Finally, CMOS electrode array was used to investigate electrical stimulation of the SGN.

## Materials and Methods

### Complementary semi-conductor metal oxide (CMOS) chip design

**Figure 1:**
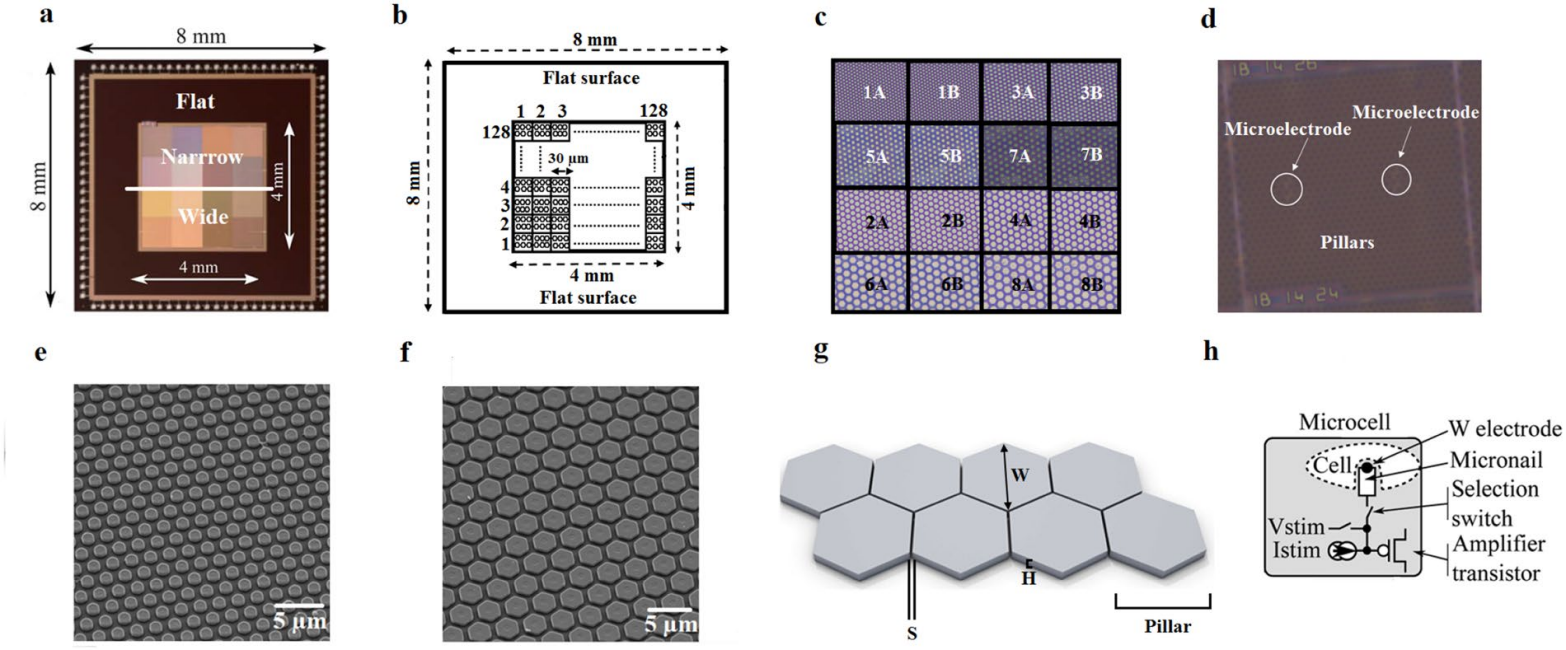
CMOS design. (**a**) Photograph of the CMOS chip. (**b**) Chip sketch. The large active area of CMOS chip measuring 4 by 4 mm consists of 128 × 128 addressable units called microcells. Each microcell is 30 by 30 µm and contains a local circuit and matrix of hexagonal micro-pillars (dummy pillars) and one active TiN micro-electrode for electrical stimulation and one recording TiN micro-electrode; (**c**) Chip pillar areas with different pillar widths (white and black numbers). A and B pillars areas have the same properties, except that micro-electrodes in area A are nail-shaped and micro-electrodes in B area are flat-shaped. Odd numbers (white, 1, 3, 5 and 7) are areas with narrow pillars: area 1: 1.4 µm; area 3: 1.6 µm; area 5: 2 µm and area 7: 2.4 µm, while even numbers (black, 2, 4, 6 and 8) are areas with wide pillars: area 2: 2.8 µm; area 4: 3.2 µm; area 6: 4 µm and area 8: 4.8 µm. The height of all pillars and electrodes is 1 µm while spacing ranges from 0.8 to 1.6 µm (0.8, 1, 1.4 and 1.6 µm) for both, narrow and wide pillars. (**d**) Microphotograph of one microcell of the chip with micro-pillars and micro-electrodes (white circles with arrows). The scale bar is 100 µm; (**e**) and (**f**) The SEM images of the CMOS surface with narrow and wide pillars respectively. The scale bar is 5 µm; (**g**) Sketch of the pillars with W and H indicating width and height of the pillar, respectively, while S indicates spacing between pillars; (**h**) One microcell of the chip is illustrated, containing the *in situ* circuits for voltage and current stimulation, an amplification transistor and the micro-nail electrode with cell position on the top of the electrode.

In this study, we used the CMOS chip with the design, fabrication and specifications reported in Huys *et al*.^35^. Briefly, the 0.18 µm TSCM platform technology was used to create probe-card wafers, consisting of a field dielectric layer of 400 nm SiO2 and one aluminum metal interconnect layer of 800 nm and 400 nm on top. Formation of three-dimensional structures with electrodes was supported by introducing vias and a layer of 100 nm TiN coating as well as with etching the oxide layer around the electrodes. In this way, an 8 × 8 mm^2^ chip array with 16,184 electrodes with individual addressability for electrical stimulation and recording was obtained. Figure 1 shows CMOS chip design together with scanning electron micrographs of superficial areas of an electrode array at different scales. Chips were grouped into two categories, depending on pillar widths. Narrow pillar widths were from 1.4 µm to 2.4 µm (1.4, 1.6, 2 and 2.4 µm) while wide pillar widths were from 2.8 µm to 4.8 µm (2.8, 3.2, 4 and 4.8 µm). Pillar spacing was from 0.8 µm to 1.6 µm (0.8, 1, 1.4 and 1.6 µm) and was equally distributed in areas of both narrow and wide pillars. Pillar height was kept constant at 1 µm. Chips were fabricated at Imec vzw (Leuven, Belgium).

### Preparation of CMOS chips and glass coverslips

CMOS chips were cleaned overnight with acetone (Gram-Mol, Zagreb, Croatia). All substrates were sterilized in 70% ethanol (Carlo Garba Reagents, Val de Reuil, France) and coated with 0.01% poly-L-ornithine (Sigma-Aldrich, St. Louis, Missouri, US) at room temperature overnight, cleaned with sterile water and allowed to dry under sterile conditions. All samples were then placed in 24-well plates (TPP, Trasadingen, Switzerland) for testing.

### SGN isolation

The University of Split School of Medicine Animal Care and Use Committee as Veterinary and Food Safety Office of the Ministry of Agriculture of Republic of Croatia approved all protocols and experimental plans used in the study (Class.003-08/15-03/0001). SGNs were isolated from 5 to 7 day-old rat pups (Sprague-Dawley) and adult guinea pigs (male and female, 350–430 g, Dunkin-Hartley). Rat pups were placed on ice, anesthetized then decapitated. Dissecting buffer contained phosphate buffered saline (PBS) supplemented with 0.3% bovine serum albumin (BSA, Sigma-Aldrich, St. Louis, Missouri, US) and 0.6% glucose (Merck, Kenilworth, New Jersey, US). Under an operating microscope, the skull was opened along the mid-sagittal plane and the brain removed. The temporal bone was harvested and transferred to clean dissecting buffer. The otic capsule was dissected and the cochlea was identified and isolated. The organ of Corti and modiolar cartilage were removed and the spiral ganglia collected in the dissecting buffer. Guinea pigs were anesthetized by intraperitoneal injection of ketamine (150 mg/kg, Richter Pharma, Wels) and xylazine (10 mg/kg, Alfasan, Woerden, Netherlands) then decapitated. The skull was bisected and the brain removed. The temporal bone was harvested and transferred to clean dissecting buffer in such way that it was rested on the external acoustic meatus. The opening in the bulla was made using fine forceps. Using this hole, the rest of the roof of the middle ear cavity was removed with a pair of a fine forceps. The bony cochlea appeared in view when the cavity of the middle ear was fully opened. With delicate movements, the spiral lamina was isolated from the rest of the bony cochlea. The modiolus became exposed and separated from the rest of the temporal bone. Following its removal, the modiolus was broken up into several small pieces using fine forceps and collected in the dissecting buffer.

### SGN cultivation

For dissociation, 500 µl of 0.25% trypsin-EDTA (Sigma-Aldrich, St. Louis, Missouri, US) with the addition of 38 U/ml DNase (Sigma Aldrich, St. Louis, Missouri, US) was used. Dissociation was performed at 37 °C for 30 minutes. The trypsinization was stopped by adding an equal volume (500 µl) of DMEM:F12 (Gibco, Waltham, Massachusetts, US) supplemented with 10% of Fetal Bovine Serum (FBS, Sigma-Aldrich, St. Louis, Missouri, US), also termed the STOP solution. For adult SGNs, the trypsinization was repeated twice for better dissociation. The tissue was then triturated, first with a 1000 µl pipette tip (gentle up and down suction for 20–25 times), followed by a 200 µl pipette tip (gentle suction for 20–25 times). Large pieces were allowed to settle down and the cell suspension was collected. The cell suspension was then centrifuged for 5 minutes at 1000 rpm and the pellet was resuspended in 500 µl of culture medium which consisted of Neurobasal-A (Gibco, Waltham, Massachusetts, US) supplemented with 1% Pen/Strep (Lonza, Basel, Switzerland), 0.25% L-Glutamine (Milipore, Billerica, Massachusetts, US), 2% B27-supplement (Gibco, Waltham, Massachusetts, US) and 30 ng/ml GDNF (Milipore, Billerica, Massachusetts, US). For the adult SGNs, centrifugation was performed for 5 minutes at 2000 rpm and the pellet was resuspended in the same culture medium where two additional growth factors were added: 30 ng/ml BDNF (Milipore, Billerica, Massachusetts, US) and 30 ng/ml NT-3 (Milipore, Billerica, Massachusetts, US). Cells were counted using a Bürker-Türk chamber and seeded in a 100 µl volume at a density of 20,000 cells/well. Cells were allowed to settle down for 1–2 h in the incubator (37 °C, 5% CO_2_, 85% humidity) when they were cultivated on glass coverslips and for 2–3 h when they were cultivated on CMOS chips, after which the rest of the medium was carefully added to the well. Half of the medium was changed every 2–3 days.

### Immunocytochemistry, cell imaging and scanning electron microscopy (SEM)

SGNs were cultured during 1, 4 and 7 days *in vitro* (DIV) and fixed with 4% paraformaldehyde for 30 minutes. For immunocytochemical analyses, samples were washed three times with 0.01 M PBS, permeabilized with 0.1% Triton X-100 (Milipore, Billerica, Massachusetts, US) for 5 minutes and blocked with PBS containing 1% normal goat serum (Dako, Glostrup, Denmark) for 90 minutes. The cells were then incubated in dark humidity chamber overnight at 4 °C in PBS with 1% normal goat serum and mouse monoclonal anti-ßIII-tubuline (1:500, Milipore, Billerica, Massachusetts, US) to stain neurons (named Tuj) and rabbit polyclonal anti-S100 (1:500, Sigma-Aldrich, St. Louis, Missouri, US) to stain glial cells. Type I neurons compose ≈95% of the spiral ganglion and innervate the inner hair cells, while Type II neurons, which compose only ≈5% of the ganglion, innervate the outer hair cells^38,39^. We used peripherin-specific (an intermediate filament) immunolabelling in order to differentiate Type II SGNs from Type I SGNs in guinea pigs, as Type II SGNs showed more powerful peripherin positivity than Type I SGNs^40–42^. Rabbit polyclonal anti-peripherin (1:200; Milipore, Billerica, Massachusetts, US) was used in combination with Tuj (1:500). On the next day, cells were washed three times with 0.01 M PBS, incubated for 90 minutes at room temperature in PBS with 1% normal goat serum with Alexa 488 goat anti mouse (1:500, Life Technologies, Carlsbad, California, US) and Alexa 568 goat anti-rabbit (1:500, Life Technologies, Carlsbad, California, US) secondary antibodies. At the end, DAPI (Molecular Probes, Eugene, Oregon, US) in a ratio of 1:500 for 5 minutes was added. The samples were finally washed five more times with 0.01 M PBS and prepared for imaging with Immu-Mount (Thermo Scientific, Waltham, Massachusetts, US). Imaging was carried out with the Olympus BX51 fluorescence microscope (Shinjuku, Tokyo, Japan) equipped with an Olympus DP71 camera. For SEM imaging, samples were fixed with 4% paraformaldehyde for 30 minutes, washed three times with 0.01 M PBS followed by washing three times in distilled water (5–10 min each time) and immersing in increasing concentrations of ethanol (steps: 25% EtOH for 5 min; 50% EtOH for 10 min; 70% EtOH for 10 min; 96% EtOH for 10 min and 100% EtOH for 10 min, three times) and then brought to the critical point of drying under air flow. A sputter coat of a thin layer of Au on top of the samples was added prior to SEM. Samples were visualized with a MIRA3 scanning electron microscope (TESCAN, Brno, Czech Republic).

### Presence and survival rate of SGNs

Given that the geometry of surface pillars exerts influence on *in vitro* grown neuronal cultures^27^, the presence and survival rates of SGNs on CMOS were analyzed across three types of areas depending on micro-pillar widths: the narrow pillar CMOS group (“Narrow”) contains neurons grown in areas with narrow pillars (1.4–2.4 µm), the wide pillar CMOS group (“Wide”) have neurons grown in areas with wide pillars (2.8–4.8 µm) whereas the flat CMOS group (“Flat”) contains neurons grown in areas of CMOS with no pillars. The number of SGNs for each experiment was calculated for each CMOS group and then the SGN presence on glass coverslips and CMOS chips was assessed and compared between different CMOS groups and a control. To quantify the total neuronal cell number, Tuj+ cells were counted under the microscope on each sample and the numbers were converted into cell densities representing the relative number of cells per mm^2^ with areas normalized by sample surface areas (8 mm^2^ for each area of a CMOS chip with micro-pillars, 132 mm^2^ for a control glass coverslip and 48 mm^2^ for the flat surface of the CMOS chip). The survival rate was calculated as previously described^10,13^ using the ratio of glial cells/neurons and the number of SGN/sample and is given as a percentage of the initial number of seeded cells per experiment which was set as 100%.

### Morphometric analysis of SGN

To describe the effect of the CMOS chip on SGN morphology, we carried out the analysis with ImageJ software (version 1.49, NIH, Bethesda, US). We focused on the following parameters: the neurite length and interaction with glial cells, the number of sprouting and neuronal alignment, each described in following sections.

### Neurite length and interaction with glial cells

A large network of axons and dendrites of spiral ganglion neurons are non-specifically referred to as neurites when grown in culture^1,10,13,14,40^. The average neurite length was described as a function of time (1 DIV, 4 DIV and 7 DIV), and as a function of different CMOS chip areas (Narrow, Wide or Flat). Neuronal cultures seeded and grown on glass coverslips represented a control. Neurites of every SGN were taken into account and their length was measured by submitting fluorescent images into ImageJ and analyzed with a Simple neurite tracer plugin (Longair MH, Baker DA, Armstrong JD. Simple Neurite Tracer: Open Source software for reconstruction, visualization and analysis of neuronal processes. *Bioinformatics* 2011). The values were then represented in graphs with standard deviation bars. In addition, we assessed the interaction between SGNs with S100+ (glial) cells by direct overlapping or contact, both on CMOS chips and in control samples, as a function of time (1 DIV, 4 DIV and 7 DIV). We defined two categories: SGNs having neurites in overlap/contact with S100+ glial cells and SGN neurites not touching S100+ glial cells. The S100+ /SGN ratio and the number of neurites of SGNs in contact with S100+ glial cells were counted manually in fluorescent, double stained images.

### SGN sprouting and morphology

The sprouting number and its relation to neuronal morphology was determined on CMOS chips, as well as on the control glass coverslips as a function of time (1 DIV, 4 DIV and 7 DIV). The morphological features were analyzed in accordance with previous work^13,14,40^. The number of neurites was scored visually in images for each Tuj+ cells to determine whereas neurons were neurite-free, mono-, bi-, multi- or pseudo-unipolar. We did not count cells that were not clearly identified or were in clumps. The percentages of different morphologies were calculated from the total number of counted neurons.

### Neuronal alignment

SGN alignment on control glass coverslips and CMOS chips was quantified using the ImageJ Fast Fourier Transform (FFT) Oval Profile plugin (authored by Bill O’Connell, https://imagej.nih.gov/ij/plugins/oval-profile.html), as previously described^27,43–45^. Briefly, 8-bit grayscale images of Tuj+ cells with a radial feather mask were created in Adobe Photoshop CS6 software (Adobe Systems Incorporated, San Jose, US), then processed with the FFT function of the ImageJ software. The directionality of the objects in the resulting images depends on the intensity and distribution of the pixels. The Oval profile plugin performed the sum of the pixel intensities for 360 radii around the center of the FFT image. A straight line from the center to the edge of the FFT image (at angle θ) quantifies the objects oriented in that direction. The directionality of the original image is represented in plots of the sum of pixel intensities along angle θ. Images with no alignment showed constant pixel intensities across all angles, while peaks along one specific direction represented an image with pixels preferentially aligned in that direction^43–45^. The values (pixel intensities) from the analyses of the orientation of each neuron were averaged between neurons and plotted in the figure between 0° and 180°, because the FFT decomposition is symmetrical around the horizontal axis and pixel summation between 180° and 360° is unnecessary.

### Analysis of neurite width asymmetry

In each CMOS sample, we identified neurons with bipolar morphology to be analyzed for neurite width asymmetry with the following procedure: first, we submitted SEM images consisting of neurons with clear bipolar morphology into ImageJ. Then, the diameter of the soma was estimated for each identified bipolar neuron. Finally, the width of each neurite (“central” and “peripheral”) emanating from the soma was measured from the soma center at distance corresponding to three times the diameter of the soma. This procedure was repeated for every bipolar SGN identified.

### Electrical stimulation set-up and live Fluo-4-AM calcium imaging

The electrical stimulation set-up consisted of a custom printed circuit board connecting PC and the CMOS electrode array. Electrical connections of the chip were sealed with a biocompatible epoxy glue (Epotek 353ND, Epoxy Technology, Inc., Billerica, Massachusetts, US). Stimulation train pulse protocols were made in the Clampex software (version 10, Molecular Devices, LLC, San Jose, California, US) and consisted of short biphasic voltage pulses^32^, each with duration of 10 µs and voltage of 1.45 V. Cells were loaded with the calcium dye Fluo-4 AM (Invitrogen, Belgium) to be able to monitor the changes in the intracellular calcium concentration ([Ca^2+^]_i_) when cells responded to electrical stimulation. The fluorescent marker Fluo-4 AM (Invitrogen, Belgium) was brought in the cells by ester loading. The final bath concentration was 5 µg/ml. The accurate position of potentially responsive SGN cells was identified by visual inspection and we chose the electrode touching either soma or neurite of the neuron for delivery of stimulus pulse trains consisting of 10 biphasic pulses. Relative fluorescent changes in the intracellular calcium concentration ([Ca^2+^]_i_) were measured using 494 nm excitation and 516 nm emission filters of an upright Examiner microscope (Carl Zeiss, Belgium) equipped with a Hamamatsu cooled CCD camera. The fluorescent changes were measured as ΔF/F_base_, the change in fluorescence intensity relative to the baseline fluorescence intensity (F_base_) prior to each stimulation pulse train.

### Statistical analysis

Data were transferred to spreadsheets and analyzed with MedCalc (version 15.2, MedCalc Software, Ostend, Belgium) and Sigmaplot (version 12.5, Systat Software, Inc., San Jose, US). The Shapiro-Wilk test, used to test the normality of the distribution of data, confirmed a parametric distribution. For the comparison of two groups we used the Student’s t-test. Multiple comparison analysis was done using one-way ANOVA, followed by a Tukey post-hoc test. Data are shown as mean ± standard deviation (SD). We considered P value of less than 0.05 as significant. Significance was labeled as not significant, ns, (P > 0.05); *, +, # (P < 0.05); **, ++, ## (P < 0.01) and ***, +++, ### (P < 0.001). Label M in the text refers to the number of experiments, label N to the number of samples used in each experiment and label n to the number of neurons analyzed. An experiment represents one 24-well plate with all samples and one sample is one glass coverslip or CMOS chip with cells.

## Results

### CMOS chip is a favorable environment for the growth of neonatal and adult SGNs

**Figure 2:**
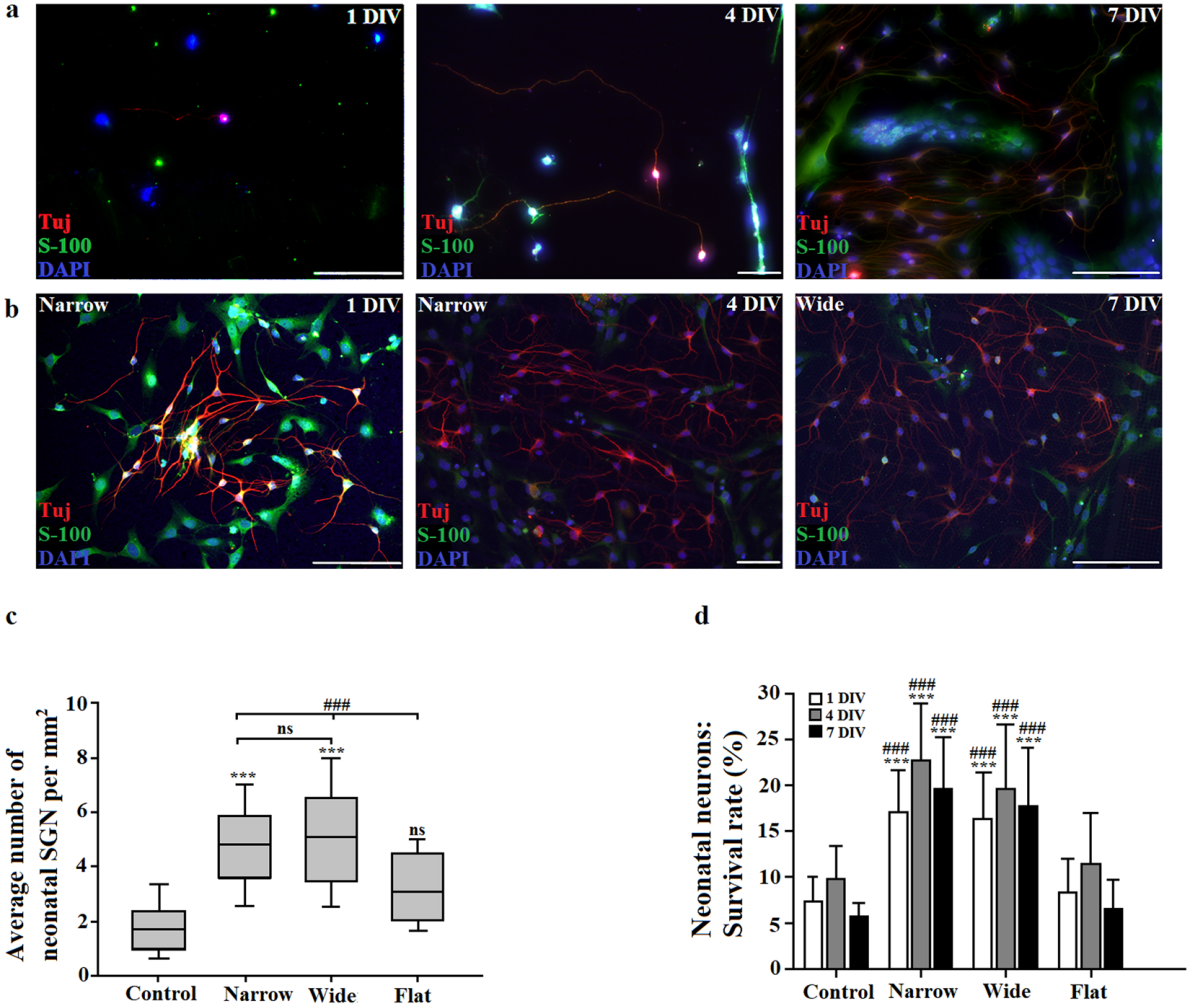
Presence of neonatal SGNs on glass coverslips (control) and CMOS chips. Neurons stained with neuronal marker Tuj+ (red/magenta) and the nuclear marker DAPI (blue). Glial cells stained with glial marker S-100 (green) and nuclear marker DAPI (blue). (**a**) SGNs on glass coverslips after 1 DIV, 4 DIV and 7 DIV. (**b**) SGN on CMOS chips after 1 DIV, 4 DIV and 7 DIV. Scale bars: 200 µm. (**c**) Box and whisker plots showing an effect of CMOS chips on neonatal SGN density. *indicates differences to control: ***P < 0.001 and ns = not significant (one-way ANOVA, Tukey post-hoc test), ^#^indicates differences between area types: ^###^P < 0.001 for the comparison between Narrow, Wide and Flat areas of the CMOS chip (one-way ANOVA, Tukey post-hoc test). (**d**) Survival rate (%) of neonatal SGN on CMOS substrates and control glass coverslips. *indicates differences to control: ***P < 0.001 (one-way ANOVA, Tukey post-hoc test) and ^#^indicates differences of Narrow and Wide areas to Flat areas of the CMOS chip: ^###^P < 0.001 (one-way ANOVA, Tukey post-hoc test).

**Figure 3:**
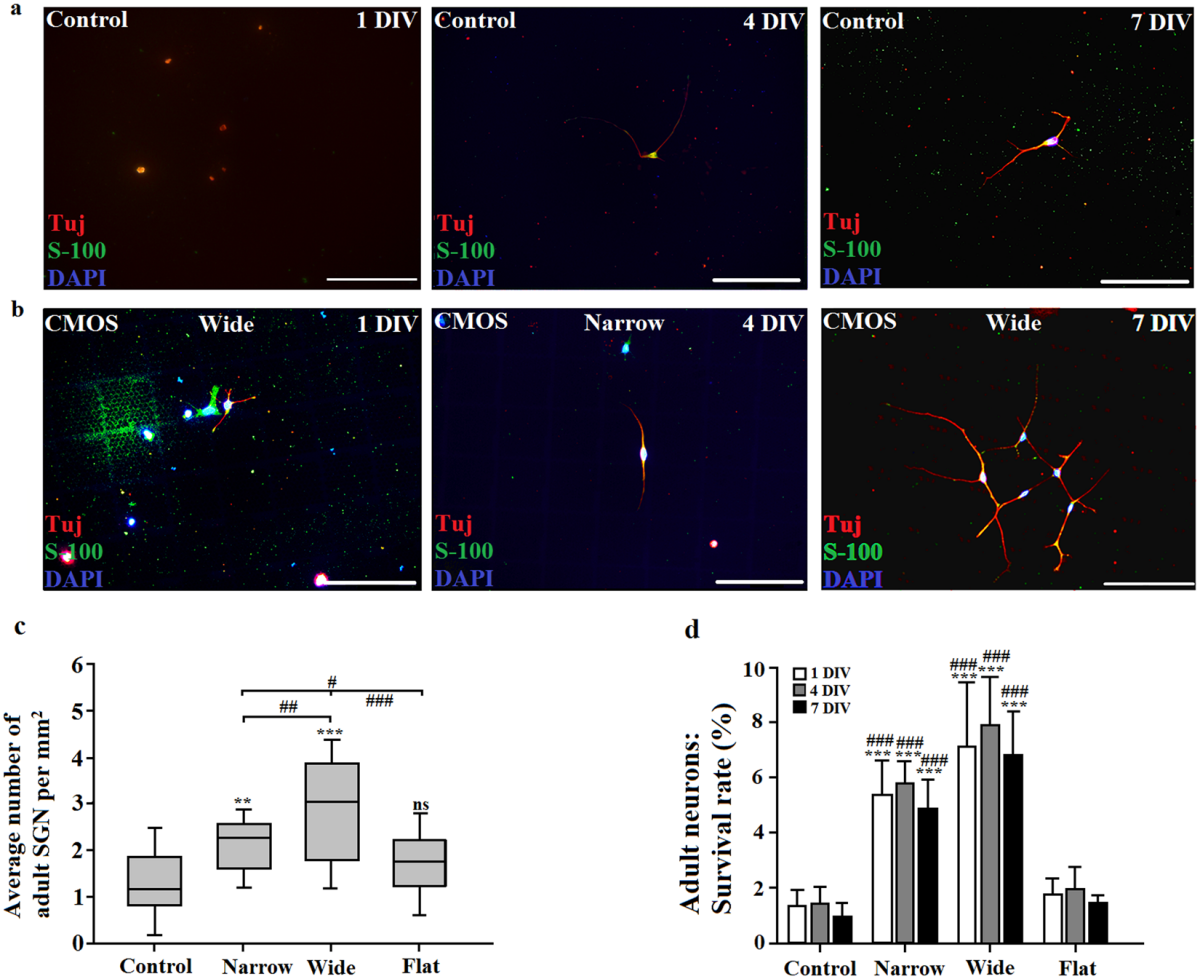
Presence of adult SGNs on glass coverslips (control) and CMOS chips. Cells were stained with the neuronal marker Tuj (red/magenta), the glial marker S-100 (green), as well as with the nuclear marker DAPI (blue). (**a**) Adult SGN on glass coverslips after 1 DIV, 4 DIV and 7 DIV. (**b**) Adult SGN on CMOS chips after 1 DIV, 4 DIV and 7 DIV. Scale bars: 200 µm. (**c**) Box and whisker plots showing an effect of CMOS chips on adult SGN density. *indicates differences to control: **P < 0.01, ***P < 0.001 and ns = not significant (one-way ANOVA, Tukey post-hoc test), ^#^indicates differences between Narrow, Wide and Flat areas of the CMOS chip: ^#^P < 0.05, ^##^P < 0.01 and ^###^P < 0.001 (one-way ANOVA, Tukey post-hoc test). (**d**) Survival rate (%) of adult SGN on CMOS substrates and control glass coverslips. *indicates differences to control: ***P < 0.001 (one-way ANOVA, Tukey post-hoc test) and ^#^indicates differences between of Narrow and Wide areas to Flat areas of the chip: ^###^P < 0.001 (one-way ANOVA, Tukey post-hoc test).

Figures 2 and 3 show an overview of the SGNs of neonatal rat pups and adult guinea pig cell cultures, respectively, on a) glass coverslips and b) a CMOS chip. Visual examination shows that neurons and non-neural cells are present both in the control and on CMOS chips with neurons growing neurites in both conditions (CMOS and control). Figure 2c shows significant differences in the average neuronal density of neonatal SGN between CMOS and control substrates. Neonatal SGN cultures on CMOS chip micro-patterned surfaces were 3-fold more dense than on control substrates (4.8 vs. 1.6 per mm^2^ respectively, Student’s t-test, P < 0.001, M = 3, N = 18 for Narrow and Wide CMOS groups. In addition, micro-patterned surfaces of CMOS chip also yielded a significantly higher density of neonatal SGNs compared to the flat zones of CMOS areas (5.1 vs. 3.2 per mm^2^, one-way ANOVA, Tukey post-hoc test, P < 0.001, M = 3, N = 18). Figure 2d shows significant differences (one-way ANOVA, Tukey post-hoc test; P < 0.001) in the survival rates between neonatal SGN grown on the different areas of the CMOS chip (Narrow, Wide and Flat) and the control glass surfaces. Survival rates of SGNs grown on the Flat surfaces of the chip and glass coverslips were similar (9.8 ± 3.4% for control vs. 11.5 ± 5.4% for Flat areas after 4 DIV) and were significantly lower, almost halved, compared to SGNs grown on the Narrow and Wide areas of the chip (22.8 ± 6.1% for Narrow areas and 19.7 ± 6.9% for Wide areas after 4 DIV). At 7 DIV, both the control and the CMOS chips yielded somewhat lower survival rates compared to 4 DIV, with the Narrow and Wide areas of the CMOS showing a smaller decrease compared to control (one-way ANOVA, Tukey post-hoc test, P < 0.05).

Figure 3a,b show an overview of the adult SGN cell culture on (a) glass coverslips and (b) CMOS chip. Figure 3c shows significant differences in the average densities of adult SGNs grown on CMOS substrates compared to control condition (one-way ANOVA, Tukey post-hoc test, P < 0.001). Adult SGN cultures on micro-patterned surfaces of the CMOS chip were about 2.4-fold more dense than cultures on glass coverslips (2.9 vs. 1.2 per mm^2^ respectively). Areas with wide pillars seem to additionally enhance SGN growth as maximal cell densities were obtained in these areas (Wide vs. Narrow areas, Student’s t-test, P < 0.01, M = 3, N = 18). We also observed differences in the neuronal density between flat areas of CMOS chips (Flat area) and micro-patterned surfaces (Narrow and Wide Zones grouped together) with Flat areas having a significantly lower density of adult SGN (2.5 vs. 1.7 per mm^2^, one-way ANOVA, Tukey post-hoc test, P < 0.001, Fig. 3c). Importantly, the control and flat areas of the CMOS chip were similar in terms of neuronal density for both neonatal and adult SGN (Figs 2c and 3c). The control group yielded survival rates of 1.4 ± 0.6% after 1 DIV, 1.4 ± 0.5% after 4 DIV and 1 ± 0.4% after 7 DIV and were comparable with the survival rates of adult guinea pig SGN growing on the flat areas of the chip (1.8 ± 0.56% after 1 DIV; 2 ± 0.74% after 4 DIV and 1.5 ± 0.23% after 7 DIV). Both were significantly lower from survival rates of SGN growing on the narrow and wide areas of the chip (5.4 ± 1.2% after 1 DIV, 5.8 ± 0.78% after 4 DIV and 4.9 ± 0.98% after 7 DIV for Narrow, one-way ANOVA, Tukey post-hoc test; P < 0.001, and 7.1 ± 2.3% after 1 DIV, 7.9 ± 1.7% after 4 DIV and 6.8 ± 1.6% after 7 DIV for Wide, one-way ANOVA, Tukey post-hoc test; P < 0.001; Fig. 3d). As for neonatal SGNs, the survival rates of adult SGN were decreased over time, and such a decrease was significantly lower on CMOS micro-patterned surface (Narrow and Wide) compared to the control (one-way ANOVA, Tukey post-hoc test, P < 0.05). When comparing neuronal densities across species, we found a significantly higher average density of neonatal SGNs compared to adult neurons (4.8 vs. 2.1 per mm^2^ respectively).

### CMOS substrates induce more bipolar and multipolar SGN morphology

**Figure 4:**
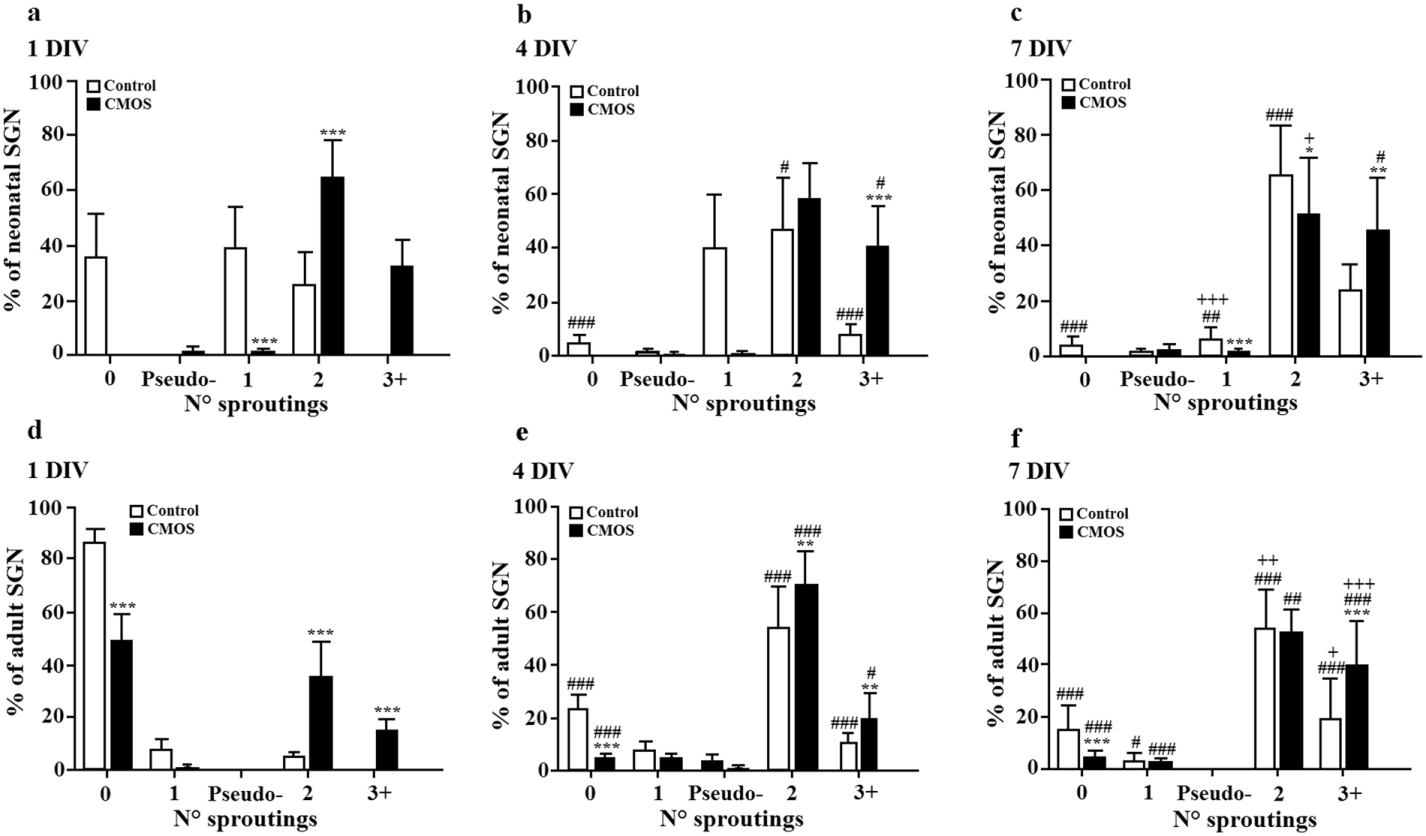
The effect of CMOS chip on neonatal and adult SGN neuronal morphology. The upper row shows data for neonatal, the lower for adult SGN, while columns separate data based on DIV time. (**a**) Quantification of neonatal SGN neuronal morphology on control and chip substrates after 1 DIV, (**b**) after 4 DIV and (**c**) after 7 DIV where N° indicates the number of sproutings (0 = neurite-free, 1 = monopolar, 2 = bipolar, 3+ = multipolar). Significant differences are indicated by # for differences by 1 DIV: ^#^P < 0.05, ^##^P < 0.01, ^###^P < 0.001 (one-way ANOVA, Tukey post-hoc test), by+ for differences by 4 DIV: ^+^P < 0.05, ^++^P < 0.01 and ^+++^P < 0.001 (one-way ANOVA, Tukey post-hoc test) and by * for differences between the control and CMOS substrates: *P < 0.05; **P < 0.01, ***P < 0.001 (Student’s t-test). (**d**) Quantification of adult SGN neuronal morphology on control and chip substrates after 1 DIV, (**e**) after 4 DIV and (**f**) after 7 DIV. Significant differences are indicated by # for differences by 1 DIV: ^#^P < 0.05 and ^###^P < 0.001 (one-way ANOVA, Tukey post-hoc test), by+ for differences by 4 DIV: ^+^P < 0.05, ^++^P < 0.01 and ^+++^P < 0.001 (one-way ANOVA, Tukey post-hoc test) and by * for differences between control and CMOS substrates: **P < 0.01, ***P < 0.001 (Student’s t-test).

The number of neurites sprouting from the SGN soma, producing neurite-free, pseudo-unipolar, monopolar, bipolar and multipolar morphologies determined the morphological shape of the SGN. As we did not observe any significant differences between Narrow, Wide and Flat areas of the CMOS chip regarding morphology, further referred to comparisons were between the control and the CMOS chip. Figure 4 shows the percentages of SGN neurons morphologically analyzed across time (columns), species (neonatal SGNs in upper panel, and the adult SGNs in the lower panel) and the area type (white bars for control and black bars for CMOS chips). Overall, bipolar neurons were the most abundant type for neonatal SGNs at all DIVs, as well as in the adults after 4 DIV. At 7 DIV, bipolar neurons were the most abundant type of neurons both in the CMOS and control conditions. Further analyses, detailed below, are performed depending on the polarity of the cells, separately for neonatal and adult neurons. First, neonatal neurite-free neurons were exceedingly more abundant in the control compared to CMOS (35.6 ± 15.9% and 0.1 ± 0.1% at 1 DIV respectively, Student’s t-test, P < 0.001; Fig. 4a). After 4 DIV and 7 DIV, there were no neurite-free neurons on CMOS. On the control coverslips, the number of neurite-free neurons decreased to 4.5 ± 2.9% after 4 DIV and to 3.8 ± 3.1% after 7 DIV. Second, in the neonatal monopolar morphology, a dramatic effect of the CMOS was observed: after only 1 DIV, the CMOS chip produced just a few neonatal SGN neurons with a monopolar morphology (1.2 ± 0.8%) representing only 1/32 fraction of the value in the control condition (38.7 ± 15.1%), with similar findings at 4 DIV (1.4 ± 0.6% on CMOS and 40 ± 19.8% on controls). After 7 DIV, percentages of monopolar neurons in the control condition was reduced by 7 times (5.8 ± 4.0%) compared to 4 DIV (40.0 ± 19.8%), and is as approximately 7 times more abundant with respect to the CMOS (0.8 ± 0.47%). Third, the CMOS seems to have a strong opposite effect on neonatal bipolar morphology: at 1 DIV, CMOS produced 2.5 times more neonatal SGN (64.6 ± 13.6%) with respect to the control (25.6 ± 12%, Fig. 4a–c). After 4 DIV, the percentages of bipolar neurons increased in the control (47 ± 19.1%) and slightly decreased on the CMOS (58 ± 13.4%), probably because the neurons developed other neurite(s) that transformed into multipolar type. Fourth, in contrast to mono- and bi- polar conditions, there were no multipolar neurons present in the control condition after 1 DIV, while the CMOS produced a high number of multipolar neurons (32.4 ± 13.3%). At 4 DIV, the percentage of neonatal multipolar SGN was still much higher on the CMOS (40.7 ± 14.9%), five times the number in the control condition (8.1 ± 3.6%, Fig. 4a–c). However, with increasing DIVs, the percentages of multipolar SGNs increased particularly on control surfaces (23.6 ± 9.4% after 7 DIV), while multipolar population on the CMOS remained fairly stable (45.4 ± 18.9% after 7 DIV).

Regarding the morphology of SGNs in adult guinea pigs, neurite-free neurons were more abundant on control surfaces compared to the CMOS (86.6 ± 4.6% vs. 48.8 ± 10% after 1 DIV, 23.2 ± 5.6% vs. 4.9 ± 1.3% after 4 DIV and 14.8 ± 9.6% vs. 4.8 ± 2.3%, after 7 DIV, Fig. 4d–f). There were no differences in the number of monopolar neurons at all DIVs between the control group and the CMOS (Fig. 4d–f). Similar to the neonatal group, bipolar morphology on the CMOS was significantly more abundant with respect to the control group at all DIVs except 7 DIV: 35.1 ± 13.9% after 1 DIV, 70.5 ± 12.7% after 4 DIV and 52.5 ± 8.7% after 7 DIV compared to control with 5.2 ± 1.8% after 1 DIV, 54.2 ± 14.8 after 4 DIV and 53.9 ± 15.2% after 7 DIV. Similar to the neonatal neurons, no multipolar adult neurons after 1 DIV on control glass coverslips were observed, while on CMOS chips, 15.3 ± 4.2% of the adult SGN were of the multipolar type. At 4 DIV, the percentages of multipolar neurons on CMOS and control surfaces were similar (Fig. 4d–f), with 7 DIV again showing more multipolar neurons on CMOS compared to the control. These results suggest that CMOS environment dramatically influenced neuronal morphology, strongly favoring bipolar and multipolar morphology, and to a less extent monopolar morphology both in neonatal and adult spiral ganglion neurons.

### CMOS micro-patterned surfaces induces elongation of a neurite and decrease neurite-glial cells interaction in neonatal and adult SGN

**Figure 5:**
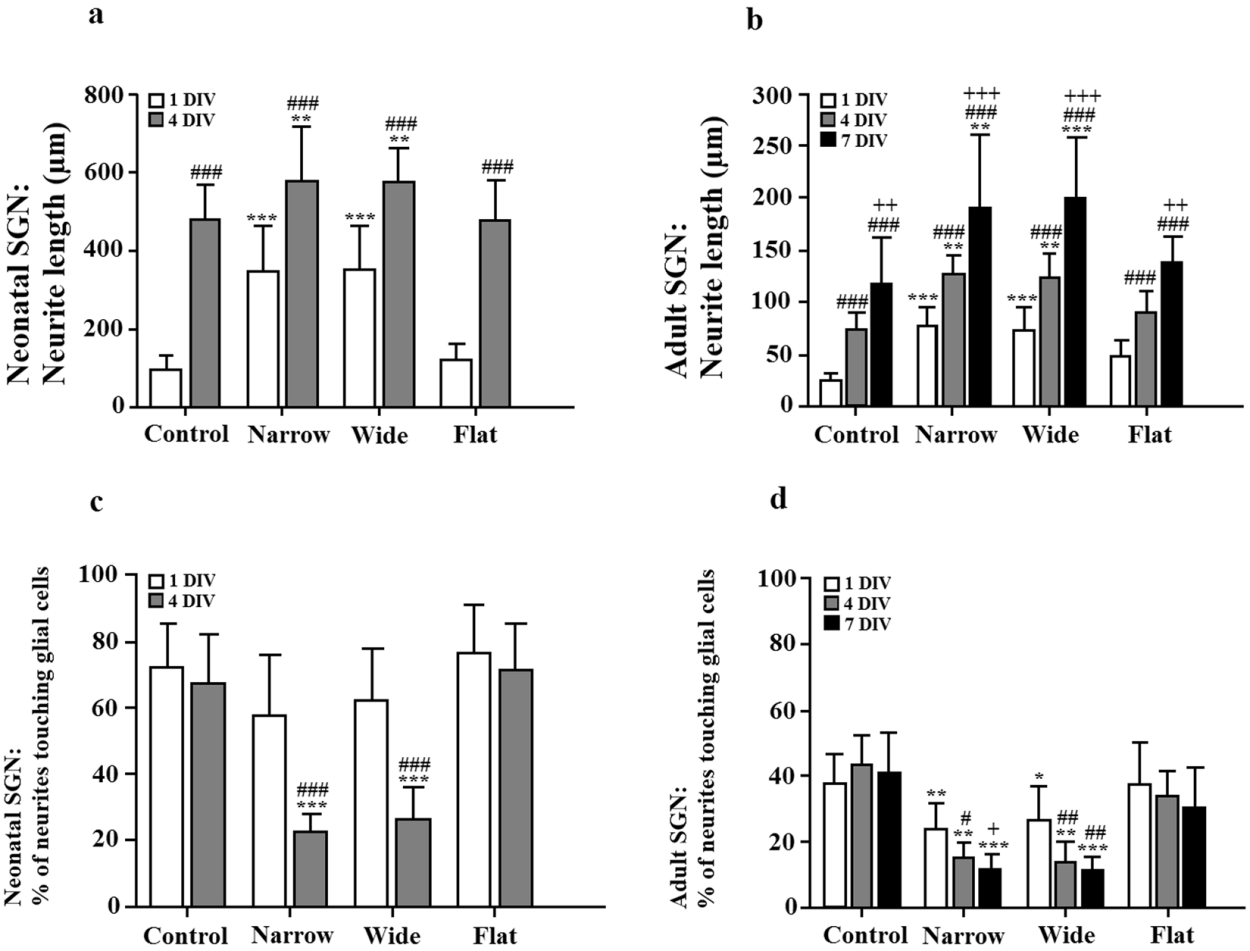
The effect of the CMOS area types on the neurite length and interaction with S100+ cells. (**a**) Bars and standard deviations show distribution of neonatal SGN neurite length as a function of time. ^#^indicates differences by 1 DIV: ^###^P < 0.001 (Student’s t-test) and *indicates differences to control: **P < 0.01, ***P < 0.001 and ns = not significant (Student’s t-test). (**b**) Bars and standard deviations show the distribution of adult SGN neurite length as a function of time. ^#^indicates differences by 1 DIV: ^###^P < 0.001 (one-way ANOVA, Tukey post-hoc test), ^+^indicates differences by 4 DIV: ^++^P < 0.01, ^+++^P < 0.001 and *indicates differences to control: ***P < 0.001 and ns = not significant (one-way ANOVA, Tukey post-hoc test). (**c**) Quantification of the interaction of the neonatal SGN neurite with S100+ glial cells in the control condition and CMOS chips as function of time. ^#^indicates differences by 1 DIV: ^###^P < 0.001 (Student’s t-test) and *indicates differences to control: ***P < 0.001 (Student’s t-test). (**d**) Quantification of the interaction of the adult SGN neurite with S100+ glial cells on control and CMOS chips. *indicates differences to control: *P < 0.05, **P < 0.01, ***P < 0.001 (one-way ANOVA, Tukey post-hoc test), ^#^indicates differences by 1 DIV: ^#^P < 0.05 and ^##^P < 0.01 (one-way ANOVA, Tukey post-hoc test) and ^+^indicates differences by 4 DIV:+ = 0.05 (one-way ANOVA, Tukey post-hoc test).

Already after 1 DIV, the neurites of neonatal SGNs on the CMOS were 3.6 times longer compared to the control group (347.6 ± 115.5 µm vs. 95.6 ± 37 µm respectively, Student’ s t-test, P < 0.001, M = 3, N = 18, Fig. 5a). The axon length was measured only at 1 DIV and 4 DIV for neonatal neurons, however, over time, the neurites from neonatal SGNs were found intertwined between each other and it was difficult to identify neurites belonging to the same cell somas. Nevertheless, in both conditions (CMOS and the control group), the neurites were significantly longer with more days *in vitro* (one-way ANOVA, Tukey post-hoc test, P < 0.001, Fig. 5a) and the increase was larger for adult neurons. There were no significant differences in the neurite length between neurons growing on narrow and wide areas of the CMOS chip for both neonatal and adult SGNs. However, the neurite length of neonatal and adult SGNs that were grown on the flat surface of the CMOS chip was similar to those on control surfaces (476.1 ± 101 µm vs. 480 ± 86.2 µm after 4 DIV for neonatal SGN and 90.4 ± 19 µm vs. 74.1 ± 16.2 after 4 DIV for adult SGNs respectively, one-way ANOVA, Tukey post-hoc test, P > 0.05, Fig. 5a,b) and was significantly lower compared to the micro-patterned surfaces of the CMOS chip (one-way ANOVA, Tukey post-hoc test, P < 0.001, Fig. 5b). Neonatal neurons had longer axons compared to adult neurons (580.1 ± 136.2 µm vs. 127 ± 17.7 µm at 4 DIV respectively). Previous observations of SGNs *in vitro* cell cultures demonstrated that these neurons often grow their neurites in contact with the S100+ cells, indicating glial type of cell^17,27,30^, most probably Schwann and satellite cells^15,27,46^. For the neonatal SGNs, the ratio of glial cells/neurons was 2.3 ± 0.6 on the CMOS chips and 3.2 ± 0.9 for the control group after 4 DIV, 5.1 ± 1.2 (CMOS) vs. 8.6 ± 3.7 (control) after 7 DIV. For the adult SGNs, the ratio of glial cells/neurons was 2.6 ± 1.0 for the CMOS chips and 3.6 ± 1.3 for the control group after 4 DIV and 2.1 ± 0.7 (CMOS) vs. 2.9 ± 1.1 (control) after 7 DIV. Furthermore, we analyzed the interaction of a neurite from neonatal and adult SGNs with the S100+ cells after 1 DIV, 4 DIV and 7 DIV for CMOS and control surfaces (Fig. 5c,d). After 1 DIV, in the control group, 72.4 ± 13.2% of the neonatal SGN neurites and 37.6 ± 9.1% of the adult SGN neurites sprouting from the somas were in contact with the S100+ cells which was not significantly different from the micro-patterned surfaces of the CMOS chip where 57.6 ± 18.4% of the neonatal neurites and 24.1 ± 7.6% of the adult neurites were touching the glial cells. (One-way ANOVA, Tukey post-hoc test, P > 0.05). After 4 DIV, in the control group, 67.4 ± 14.6% of the neurites sprouting from the neonatal SGN soma were in contact with the glial cells, while on the micro-patterned surfaces of the CMOS chip this percentage was almost 3 times lower (22.9 ± 5.1% for Narrow Pillar areas and 26.7 ± 9.7% for Wide Pillar areas, one-way ANOVA, Tukey post-hoc test, P < 0.001). Adult SGNs growing on micro-patterned surfaces of the CMOS chip had 15.5 ± 4.6% (Narrow Pillar) and 14.1 ± 5.8% (Wide Pillar) neurites in contact with glial cells after 4 DIV, which was significantly lower compared to the control (43.4 ± 8.6%) and flat surface of the chip (34.1 ± 7.4%, one-way ANOVA, Tukey post-hoc test, P < 0.001). A similar outcome was also found after 7 DIV for adult SGNs, since neonatal neurons were difficult to analyze.

### Specific CMOS topography elicits directional SGN neurite orientation and alignment

**Figure 6:**
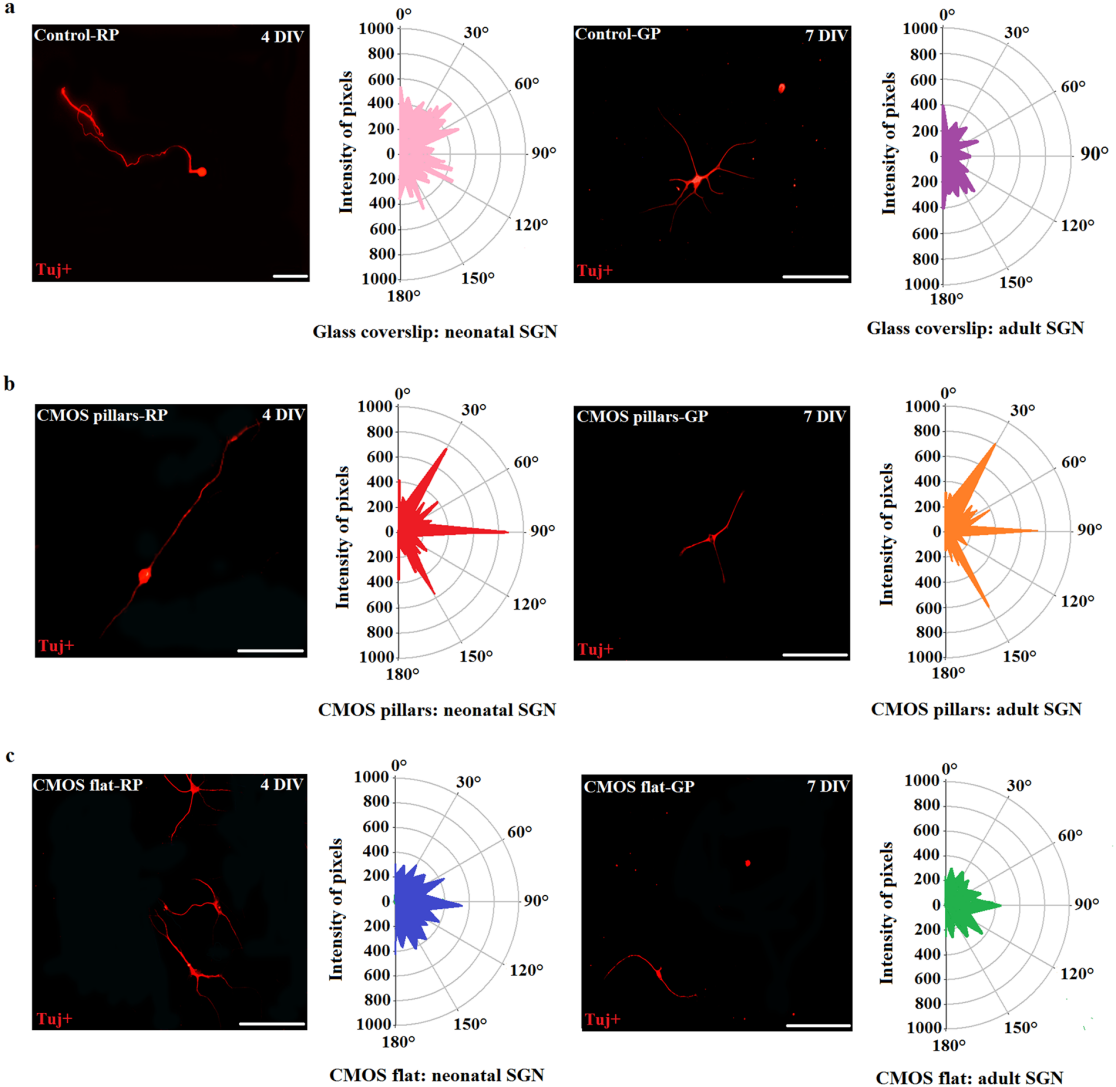
The effect of CMOS topography on neurite orientation and alignment. Representative fluorescent images of Tuj+ neonatal and adult SGN alignment as well as alignment profiles of neurite growth around the preferred angles on glass coverslips (**a**), CMOS micro-patterned surfaces (**b**) and CMOS flat surfaces (**c**). RP = rat pups, GP = guinea pigs. Scale bars: 200 μm. The radial values range from 0–1000 representing the intensity of pixels from FFT images along the same angle, while the angles are from 0 to 180 degrees. The values obtained by FFT Oval Profile picture analysis were averaged across neurons. For the control, 77 neonatal and 42 adult SGNs were analyzed. (N = 18), For CMOS micro-patterned surfaces, 203 neonatal and 162 adult SGNs were analyzed (N = 18) and for CMOS flat surfaces, 134 neonatal SGN and 87 adult SGNs were analyzed (N = 18).

In order to determine how micro-pillars dimensions (width and spacing) of the CMOS chips influenced the neurite orientation and alignment, we stained and visualized neonatal and adult SGNs grown on CMOS chips and glass coverslips with Tuj+ (Fig. 6). The FFT Oval Profile analysis showed that the hexagonal topographical pattern of the pillars had a strong influence on the alignment and orientation of neurite outgrowth on CMOS micro-patterned surfaces (Fig. 6b). On specific micro-patterns, neurites were shown to be preferentially oriented and aligned along three directional axes, spaced by 60° angle intervals (30°; 90°; 150°), closely following angles within hexagon. On the other hand, the topographic guidance was rather reduced on glass coverslips (control) and flat areas of the chip (Fig. 6a,c), suggesting that only specific topographic structures of the CMOS substrates provide mechanical support and guidance for growth and differentiation of SGNs. Pillars width was shown not to have influence on neuronal alignment and orientation, since the effect of pillar widths was similar in narrow and wide areas of the CMOS substrates.

**Figure 7:**
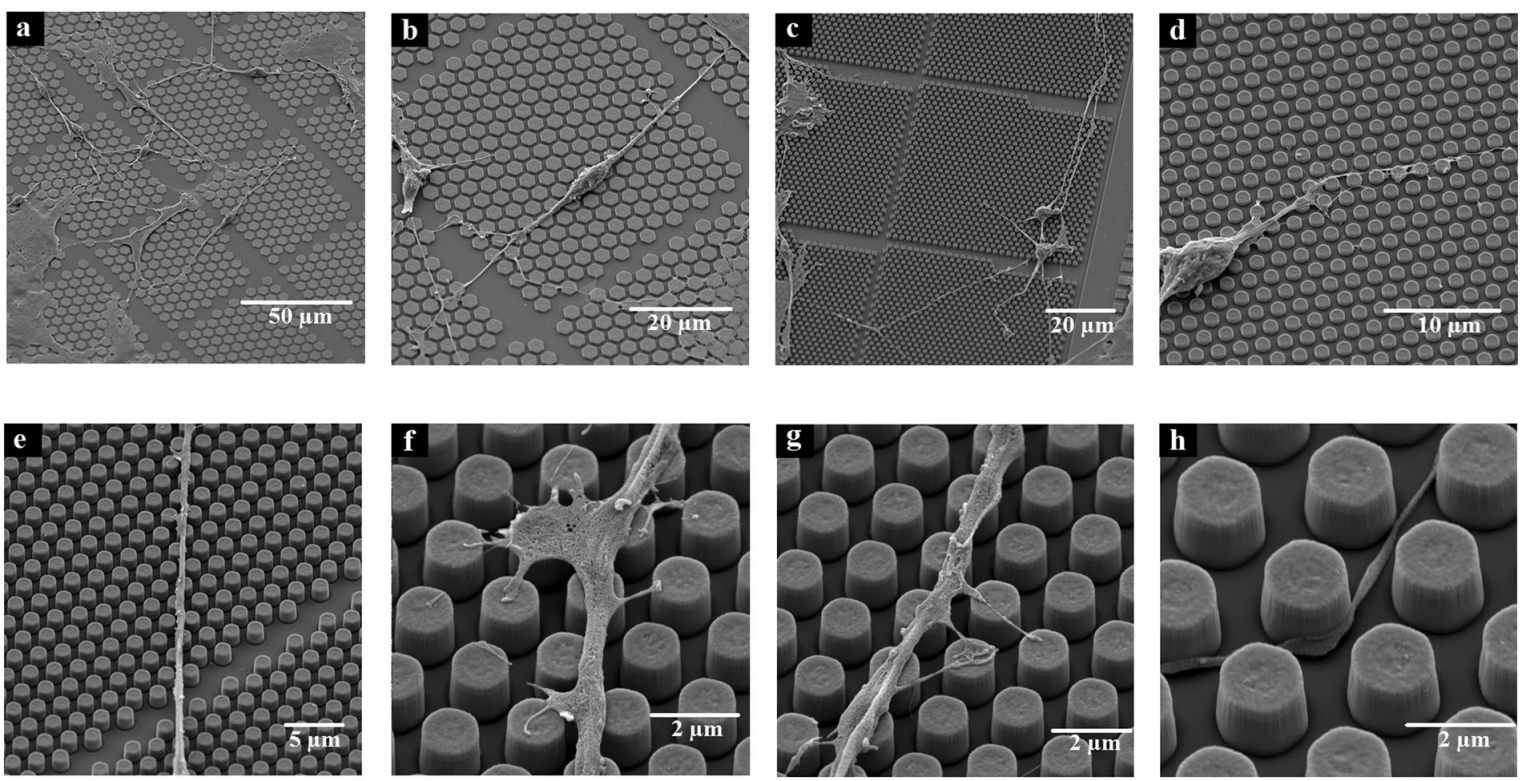
SEM images of spiral ganglion neurons cultured on CMOS chips. (**a**) SGNs cultured on CMOS chip. (**b**) Bipolar neuron cultured on CMOS chip. Neurites grow in straight lines. (**c**) Two neurons grow on top of the CMOS chips without the favorable flat surface of the chip. (**d**) SGN cell soma with the neurite following micro-pillars on the CMOS chip. (**e**) The neurite is guided by micro-patterned surface of the chip, follows a single angle determined by the spatial distribution of the pillars and can cross a small divide of 2 µm without pillars between microcells (see Fig. 1) and attaches to the pillars on the other side of the divide; (**f**) Growth cone formation on micro-pillars of the CMOS chips serves for axon repositioning; (**g**) One neurite with protruding branches seeking interaction with pillars; (**h**) one neurite passing between pillars showing how it touches pillars, probably in order to maintain the contact guidance.

To visualize neurons growing on the CMOS areas with micro-patterned pillars, we obtained scanning electron images (SEM) of the spiral ganglion neurons after cultures were properly stained and prepared. Figure 7 shows the interaction of neurites with pillar structures and micro-electrodes. Neurites grew in straight lines on top and between pillars in mostly a single direction with occasional perpendicular branching. Neurites on pillars also formed a nerve growth cone which filopodia utilize as anchoring points for axon repositioning when on the CMOS pillar.

### CMOS topography promotes growth of Type I and Type II of adult SGNs

**Figure 8:**
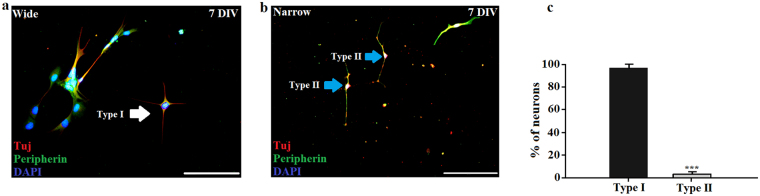
Presence of Type I and Type II of adult SGNs. (**a**) and (**b**) Fluorescent images of guinea pigs type I and II SGNs on CMOS chips: Neurons stained with neuronal marker Tuj (red) and the nuclear marker DAPI (blue). Type II SGNs stained with peripherin (green) and the nuclear marker DAPI (blue). The white arrow shows Type I SGNs and blue arrows show Type II SGNs from guinea pigs. Scale bars: 200 µm. (**c**) Percentage of Type II SGNs from guinea pigs in comparison with Type I SGNs (M = 3, N = 18, n = 386). ***P < 0.001 (Student’s t-test).

Adult SGNs cultured on CMOS chips and glass coverslips were stained with rabbit polyclonal anti-peripherin to distinguish between Type I and Type II SGN. While there were no anti-peripherin positive neurons in the control condition, the CMOS substrates contained 3.3 ± 1.9% of neurons (n = 386, M = 3, N = 18) showing a strongly positive effect for anti-peripherin (Fig. 8b,c). The rest of SGNs were not strongly positive for anti-peripherin and were identified as Type I SGNs (Fig. 8a,c). The average diameter of somas from Type II SGNs was 11.4 ± 1.32 μm and was significantly smaller than the somas from Type I SGNs (23.6 ± 7.8 µm, Student’s t-test, P < 0.001). The average length of a neurite was 137.9 ± 26.9 μm and was also significantly shorter than the length of a neurite from Type I SGNs (200.3 ± 56.7 µm, Student’s t-test, P < 0.001). Type II SGNs can be bipolar or pseudounipolar^38,39,47^. In this study, mostly bipolar Type II SGNs appeared on the CMOS chip (Fig. 8b).

### Micro-patterned surfaces of the CMOS chip represent a favorable environment for electrophysiological applications

**Figure 9:**
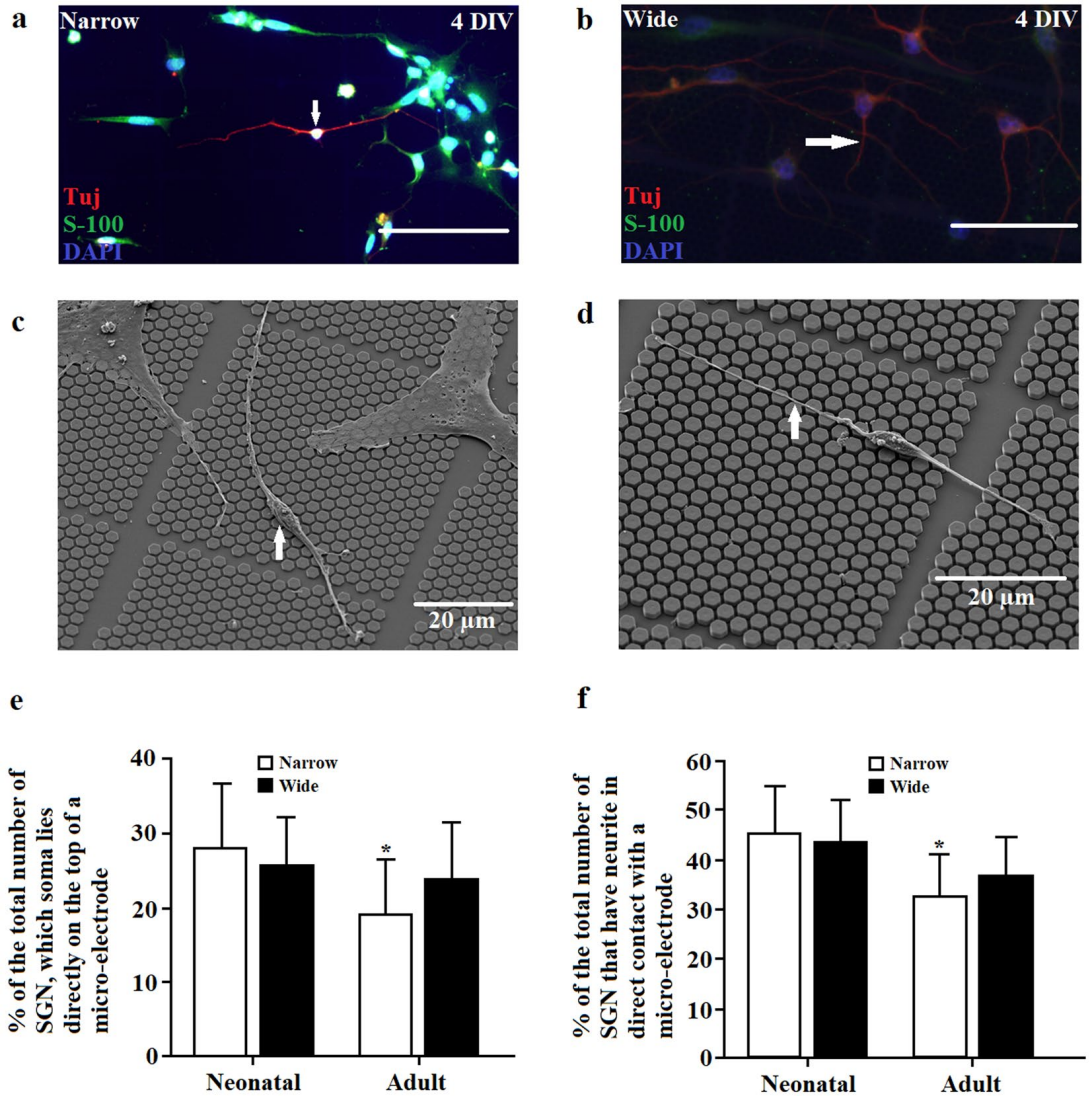
Contacts of the SGN soma and neurites with micro-electrodes. (**a**) Fluorescent image of a neonatal SGN with its soma being positioned directly on the top of a micro-electrode (white arrow). Cells were stained with the neuronal marker Tuj (red/magenta), glial marker S-100 (green), as well as with the nuclear marker DAPI (blue). Scale bar: 200 µm. (**b**) Fluorescent image of a neonatal SGN with a neurite spanning a micro-electrode (white arrow). Cells were stained with the neuronal marker Tuj (red/magenta), glial marker S-100 (green), as well as with the nuclear marker DAPI (blue). Scale bar: 200 µm. (**c**) SEM image of a neonatal SGN with its soma positioned directly on the top of a micro-electrode (white arrow). (**d**) SEM image of a neonatal SGN with neurite spanning a micro-electrode (white arrow). (**e**) Quantification of neonatal and adult SGNs with their somas positioned directly on the top of a micro-electrode. *indicates difference between neonatal and adult SGNs on narrow pillar area: *P < 0.05 (Student’s t-test). (**f**) Quantification of neonatal and adult SGNs with neurites spanning a micro-electrode. *indicates difference between neonatal and SGNs on narrow pillar area: *P < 0.05 (Student’s t-test).

The position of the micro-electrodes on the chip and spacing between pillars are parameters that affect electrophysiological measurements. Recordings of electrical activity and electrical stimulation on SGNs can presumably be performed if their soma lies directly on the top of a micro-electrode or if their neurites are in direct contact with a micro-electrode. Figure 9 shows that 28 ± 8.7% of the total neonatal SGNs growing on Narrow areas and 25.7 ± 6.3% of the total neonatal SGNs growing on Wide areas had their soma positioned on the top of a micro-electrode. Similarly, 23.9 ± 7.6% of the total adult SGNs growing on Wide pillars and 19.2 ± 7.1% of the total number of adult SGNs growing on Narrow areas had their soma positioned on the top of a micro-electrode. Comparing across species and CMOS areas, we found that only the Narrow areas of CMOS chips yielded a significantly higher number of neonatal SGNs with their soma residing on the top of the micro-electrode (Student’s t-test, P < 0.05, M = 3, N = 18). Similar findings were observed within all days *in vitro* for both, neonatal and adult SGNs. Expanding analyses with neurites going over the micro-electrode, we found that neonatal SGNs had 45.2 ± 9.2% of the total neurites growing on Narrow areas are in direct contact with a micro-electrode and 43.2 ± 8.5% of the total neurites have direct contact with a micro-electrode on Wide areas. Adult SGN had 32.7 ± 8.3% of the total neurites growing on Narrow areas being in direct contact with a micro-electrode and 36.7 ± 7.5% in the case of Wide pillars, which is significant smaller compared to the neonatal SGNs on narrow pillars (Student’s t-test, P < 0.05, M = 3, N = 18). Similar results were confirmed for all of the days *in vitro* (Fig. 9e,f). These results suggest that CMOS micro-patterned surfaces provide not only a favorable environment for the growth and alignment of neonatal and adult SGN, but can also enable effective electrophysiological stimulation and recording.

### The CMOS electrode array enables electrical stimulation of the SGNs

**Figure 10:**
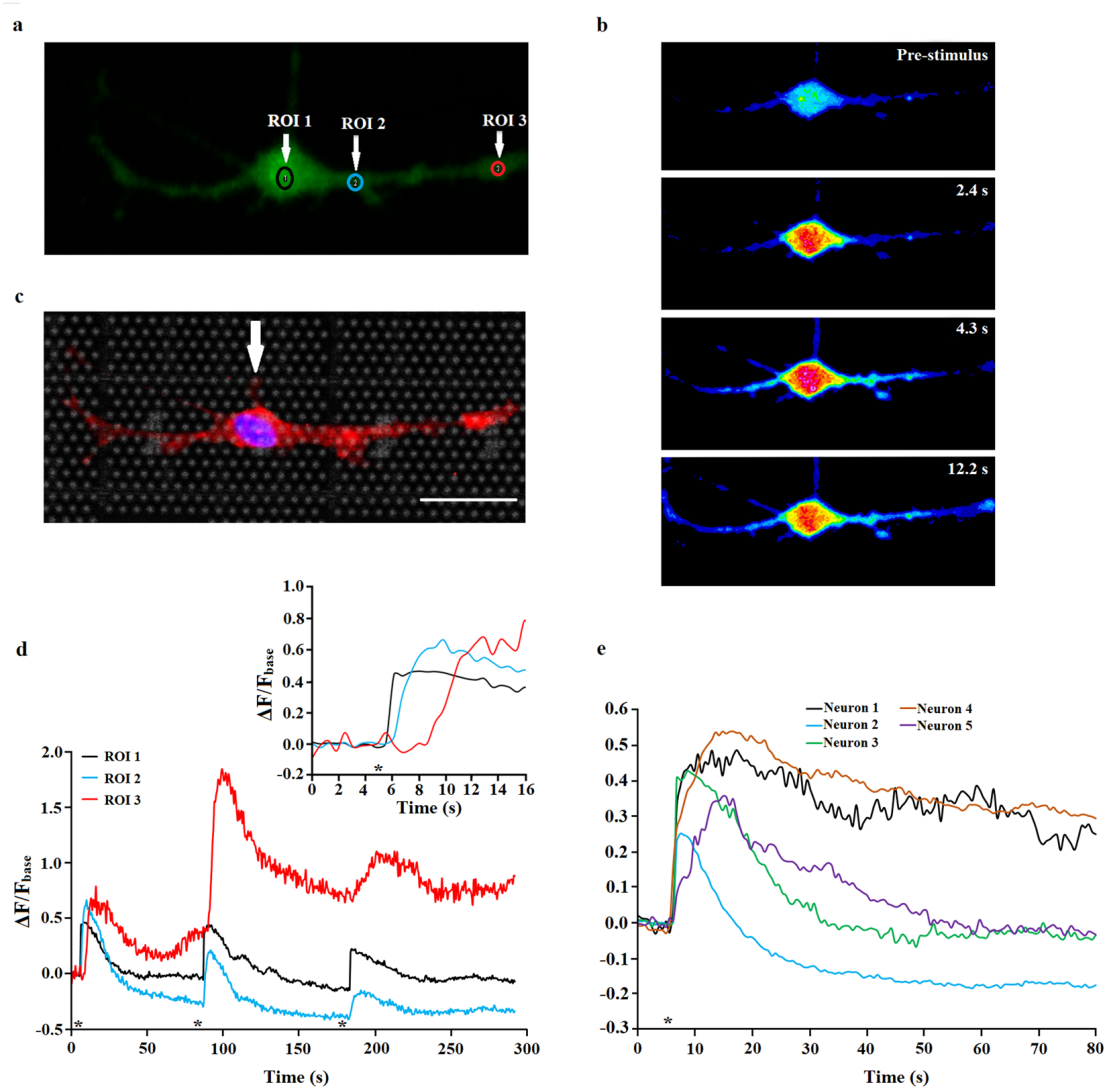
Fluo-4-AM calcium imaging and localized stimulation of SGN by biphasic electrical stimulation. (**a**) An image of a spiral ganglion neuron after Fluo4 calcium staining. Regions of interest are indicated with the yellow circles (ROI 1, ROI 2, ROI 3). (**b**) Time sequence of intracellular calcium concentration ([Ca^2+^]_i_) of a single SGN in response to electrical pulse train. Timing of the image capture is shown in upper right corner and is relative to the stimulus onset. (**c**) Fluorescent image of a spiral ganglion neuron stained with Tuj (neurons, red) and S-100 (glial cells, green). The stimulated cell was positive for neuronal marker Tuj only. The soma of the stimulated SGN lies directly on the top of the micro-electrode (white arrow). Scale bar: 25 µm. (**d**) Relative changes in [Ca^2+^]_i_ in three regions of interest indicated in (**a**) by circles with corresponding color. The inset shows a magnification of the graph right after the first stimulation. *indicates the onset of the electrical pulse train. (**e**) Relative changes in [Ca^2+^]_i_ in five observed neurons indicated with the corresponding color.

We cultured spiral ganglion neurons for 6 DIV and 7 DIV on top of the CMOS electrode array chip to investigate the ability to electrically stimulate SGNs. Neurons were loaded with the calcium dye Fluo-4-AM to be able to monitor the changes in the intracellular calcium concentration ([Ca^2+^]_i_) of the SGNs close to the stimulation electrode. We selected a pair of electrodes that was located near or beneath the presumptive neuronal soma or neurite of the neuron and applied a train of biphasic pulses between electrodes. Stimulation pulses were applied through the electrodes while monitoring the changes in [Ca^2+^]_i_. Here we report calcium imaging responses in five spiral ganglion neurons stimulated with biphasic pulse trains (Fig. 10e). Strong increase in the relative [Ca^2+^]_i_ initiated in neurons whose soma lies directly on the top of a micro-electrode or whose neurites are in a direct contact with a micro-electrode in response to electrical stimulation. Stimuli were trains of either 5 or 10 pulses, each with duration of 10 µs and stimulation voltages of 1.45 V. Figure 10a–d show calcium influx responses from a single spiral ganglion neuron 7 DIV, isolated from rat pup, with the soma positioned on top of the electrode which was stimulated with three biphasic voltage pulses, indicated with the asterisk. In Fig. 10a, we indicated three regions of interest (ROI 1, ROI 2 and ROI 3) along Fluo-4 calcium stained spiral ganglion neuron and noticed that the signal of calcium fluorescent changes spread from neuronal soma along both neurites (Fig. 10b,d - inset). The same neuron was then stained with both the neuronal marker Tuj and glial marker S-100. The stimulated cell was positive only to Tuj marker, indicating the cell was indeed a spiral ganglion neuron (Fig. 10c). The overall temporal dynamics of calcium imaging responses followed relatively similar time courses in all five observed neurons (Fig. 10e).

### Neurite width asymmetry

**Figure 11:**
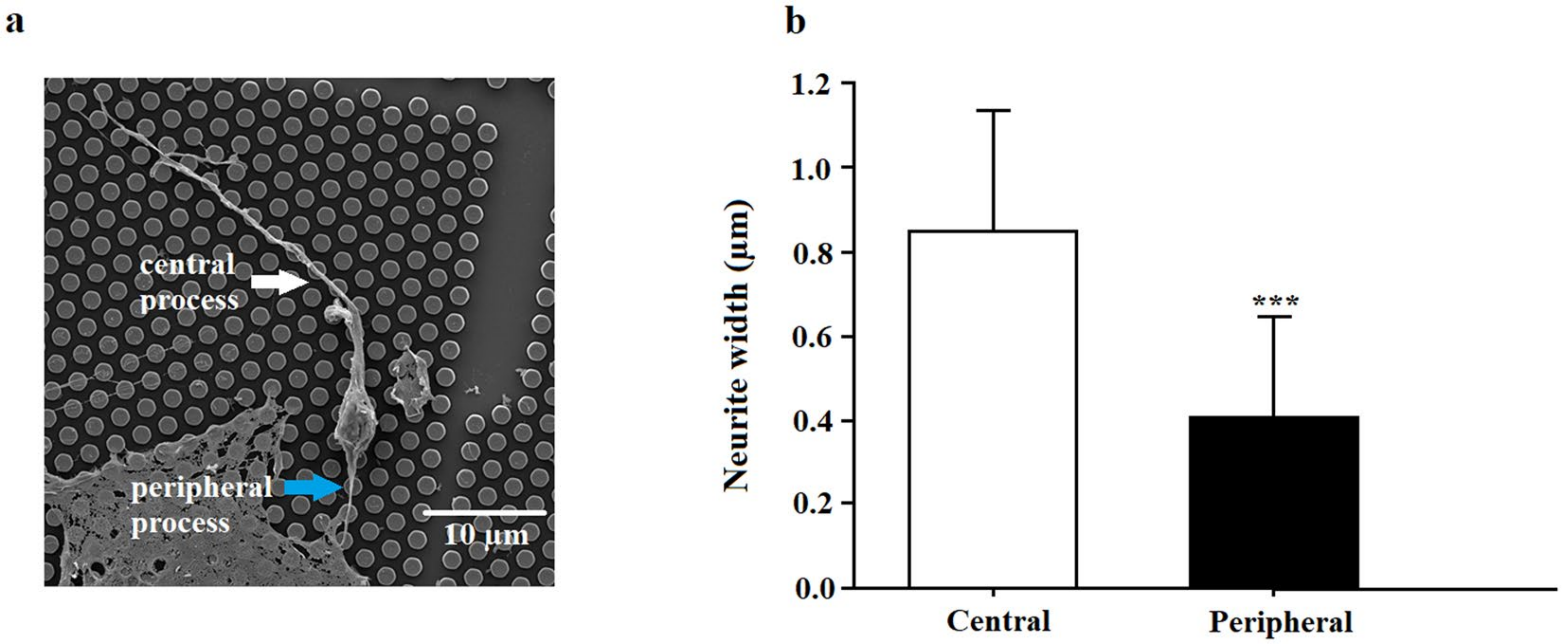
Neurite width asymmetry. (**a**) SEM image of a typical bipolar spiral ganglion neuron with its central and peripheral processes (white and blue arrow). (**b**) Quantification of neurite width: *indicates a significant difference between central and peripheral processes width: ***P < 0.001 (Student’s t-test).

The bipolar spiral ganglion soma has multiple morphological specializations, such as the proximity to surrounding nodes and the differential diameter of its central versus peripheral neurite processes. This neurite width asymmetry presumably counteracts “branch failures” which, if unchecked, would ultimately impede action potential conduction towards the central nervous system^48^. Thus, we analyzed neurite width asymmetry for each SGN with the bipolar morphology using SEM images providing clear neurite processes. Figure 11a demonstrates this phenomenon observed in the SEM image for neonatal SGNs cultured *in vitro* on the CMOS chip showing two opposite neurite processes with differential widths; the wider one presumably represents the central process and the thinner one indicates the peripheral process in accordance with the neuroanatomy of the SGNs^39^. Measuring neurite width asymmetry in all neonatal SGNs with a bipolar morphology available on SEM images, we found significantly wider widths of one neurite process compared to the opposite neurite process (Fig. 11b. the average width of thinner process (peripheral process) was 0.41 ± 0.24 µm and that of wider process was 0.85 ± 0.28 µm Student’s t-test, P < 0.001, M = 1, N = 5, n = 24.

### Micro-patterned surfaces of the CMOS chip demonstrate feasibility for normal neuronal growth without coating

**Figure 12:**
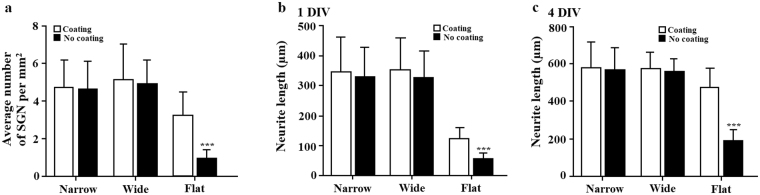
Influence of a substrate coating on neuronal presence and growth. (**a**) Bars and standard deviations showing an effect of substrate coating on SGN density. *indicates significant differences between coated and non-coated flat surfaces of the CMOS chip: ***P < 0.001 (Student’s t-test). Bars and standard deviations showing an effect of substrate coating on neurite length after (**b**) 1 DIV and (**c**) 4 DIV. *indicates differences between coated and non-coated flat surfaces of the CMOS chip: ***P < 0.001 (Student’s t-test).

To assess the extent of the advantageous environment of the micro-patterned surfaces with embedded electrodes for neuronal growth, we looked at whether non-coating of the substrate surfaces would be detrimental to neural growth. Specifically, we cultured SGNs on the CMOS chips without the addition of any coating chemicals (i.e., poly-L-ornithine) which is usually required for *in vitro* cultures. In our case, we found similar SGN presence on the chips with and without coating only on micro-patterned areas of the chips containing pillars and micro-electrodes (Fig. 12a). On the other hand, neuronal density was significantly decreased on non-coated Flat areas of the chip compared to the same surface coated with poly-L-ornithine (3.2 ± 1.2% on coated Flat areas of the CMOS vs. 0.97 ± 0.44% on non-coated Flat areas of the CMOS, Student’s t-test, P < 0.001, M = 2, N = 12). Similar to neurons, the number of glial cells identified as S100 positive cells was also decreased on non-coated Flat areas of the CMOS chip. As for neurite length, we found that neurons express similar growth patterns on non-coated micro-patterned CMOS surfaces as on coated surfaces. No significant differences were found in neurite length for coated and non-coated CMOS micro-patterned surfaces (one-way ANOVA, Tukey post-hoc test, P > 0.05, M = 2, N = 12). However, there was a significant difference in neurite length between coated and non-coated Flat areas of the CMOS; neurites on non-coated flat surfaces of the CMOS chip were almost half the length (56.7 ± 19.2 µm) when compared to coated flat surfaces (123.3 ± 37.4) after 1 DIV and 2.5-fold shorter (189.4 ± 58.6 µm) after 4 DIV in comparison to coated flat surfaces (476.1 ± 101 µm). Neuronal alignment as well as interaction with glial cells and morphology were similar on non-coated substrates as for coated substrates described above. In striking contrast with the micro-patterned CMOS surfaces, neuronal attachment to control glass coverslips without coating was negligible, demonstrating the necessity for substrate coating. These results confirm that micro-patterned surfaces with embedded micro-electrodes of the CMOS chip represent a favorable environment for neuronal attachment and growth without coating chemicals.

## Discussion

Our results demonstrate that micro-patterned and high-density CMOS based electrode array represents an advantageous and favorable environment for *in vitro* SGN cultures by demonstrating stronger cell presence of the SGN, faster neurite sprouting with strong bipolar morphological polarity, reduced neuronal-glial interactions and with the ability to electrically stimulate the SGNs. More specifically, micro-patterned surfaces of the CMOS substrates promoted neuronal growth more effectively than glass coverslips and even flat areas of the chip containing no protruding pillars (Figs 2c and 3c). These effects were similar for neonatal and adult SGN cultures. Measurements of neurite lengths highlighted longer neurites, which also implies faster sprouting on CMOS chips compared to control for neonatal and adult SGNs (Fig. 5a,b). The study by Mattotti *et al*. with neonatal SGNs on passive silicon micro-patterned surfaces, albeit with no active CMOS component shows that neurons favor pillar spacing between 1.2 and 2.4 μm for improved growth and neurite elongation^27^. Our studies further extended these findings by showing differences in neuronal distribution between neonatal and adult SGNs cultured on CMOS chips with different pillar widths and pillar spacing between 0.8 and 1.6 µm. To the best of our knowledge, this is the first study where SGNs express normal growth also on non-coated micro-patterned CMOS surfaces, which can be useful in future clinical application due to the degradation of the coating chemicals over time and safety issues. Cultures on non-coated glass coverslips poorly attached, preventing neuronal growth on non-coated substrates. The flat surfaces of the non-coated chips showed poor neuronal growth, because the neurons could not firmly attach without pillar support. These findings demonstrate that micro-patterned surfaces consisting of protruding pillars and embedded micro-electrodes serve as an advantageous environment for neuronal attachment and growth. Neonatal SGNs have shown preferential growth in areas with both narrow and wide pillars, while adult SGNs seem to prefer to grow on wide pillars. This may be the consequence of either larger soma diameter or lower plating yield of adult SGNs making preference for wide pillars in adult SGNs. Similar observation was presented in Repić *et al*. study for adult dorsal root ganglion neurons. Our data indicate that neonatal and adult SGNs on CMOS chips had the highest survival rates, whereas control glass coverslips induced weaker cell survival (Figs 2d and 3d). The best survival rate was observed at 4 DIV, for both, neonatal and adult SGNs, showing neuronal viability peaking around that time. We surmise lower survival rates at 7 DIV are due to proliferation of non-neuronal cells, such as glial cells over time. This is consistent with previous studies in which fibroblasts and glial cells proliferate but neurons do not^15,49,50^. However, such a decrease was significantly weaker on micro-patterned surfaces of the CMOS chip. Compared with other studies reporting primary SGN cell cultures, we found our survival rates to be comparable and improved in some conditions. For instance, Jin *et al*.^19^ report a P5 SGN survival rate of 0.98% after 3 DIV, Vieira *et al*.^13^ describe the survival rate of adult SGNs from rats, mice or guinea pigs ranging between 1 and 6%, Mattotti *et al*.^27^ report a P5 SGN survival rate on MPS of 4.8% and Schwieger *et al*.^10^ describe P2-P4 SGN survival rate ranging from 3.57 to 40.69%, depending on the combination of different growth factors. Thus, the results in the present study confirm that micro-patterned CMOS substrates represent more supportive and permissive environment for neuronal growth and survival, compared to glass coverslips. Topographical cues strongly promote neuronal orientation and neurite guidance^21,23–27^ which are observed in SEM images (Fig. 7) and in polar plots of neural orientation showing neurite growth along 30°, 90° and 150° radial angles, compared to the control where there was no preferred orientation of the neurites (Fig. 6). SEM images highlight how SGNs develop their neurites in straight lines on top and between pillars (Fig. 7). Only areas with pillars and micro-electrodes favor neurite alignment along preferred angles. These findings strongly suggest that pillar width and spacing significantly influenced SGN neurite orientation and alignment that is crucial for structuring neurite connectivity. Pillars and micro-electrodes represent a strong and stable adhesion milieu where neurites are extended. It seems that the protruding shape and carefully crafted three-dimensional structureof pillars behave like geometrical constraints providing directional guidance. Closely spaced pillars and micro-electrodes stimulate neuronal growth by creating boosts at their contacts with neurons, compared to more separated pillars where signaling delay occurs. Other studies using silicon MPS^28,29^ demonstrated that neurites were found to “search” for a new nearby permissive cue (pillar) and attach to areas with the largest pillar spacing, whereby this process elicits a structural change. Our SEM images also showed the formation of nerve growth cones provided cells with a mechanical apparatus that guided neurite growth to mechanical, chemical and electrical cues^51^. On micro-patterned surfaces of the CMOS chips, filopodia of the growth cone used pillars or micro-elecrodes as an anchoring point from where neuronal growth may occur. The presence of particular structures of micro-pillars and micro-electrodes on CMOS chips also strongly influenced SGN morphology. The number of neurons with bipolar and multipolar morphologies grown on CMOS substrates was stable over time while the number of neurons with monopolar and other morphologies was significantly reduced compared to the control group. Mattotti *et al*. reported that neonatal SGNs developed more monopolar and bipolar morphologies, when they were cultured on MPSs, while there was a reduction in the number of neurons with multipolar morphologies and concluded that MPSs might be more beneficial to induce neuronal morphologies more similar to *in vivo* conditions^27^. Our study with neonatal and adult SGNs cultured on CMOS chips demonstrated that CMOS substrates encouraged neurons to develop more bipolar and multipolar morphologies. *In vitro* neurite-free, mono-, bi- and multi-polar morphologies were also observed in other studies^10,13,14,27,40,52^. Different sprouting behaviors can be related to neuronal polarization and neurite pathfinding^28^. The appearance of SGNs *in vitro* may vary between studies, depending on the species of origin, the age of the animals and the cell culture protocol^27,40,52^. Khalifa *et al*. reported a drastic increase of multipolar neurons from 5% on non-structured surfaces to 86–87% on surfaces with alternating lines^53^. They stated that controlled sprouting and branching can be an essential process in the sense that additional neurites contribute in augmenting the chances that neurites reach their targets^53^. We surmise that the micro-topography of CMOS chips probably utilizes similar stimulation mechanisms that could be integrated synergistically with biochemical support in the design of optimal electrode arrays. The comparison of our results with results of other studies is difficult because of different material, size, shape and uniformity of the CMOS chips as well as different techniques used for the analysis of the alignment and neuronal behavior and measurements. *In vitro* cultures of SGNs also comprise an important presence of non-neuronal cells, mostly glial cells, where the ratio to neurons can vary, for example from 1:1 to 20:1^4,13^. However, our study suggests different ratios of glial cells/neurons. The ratio of glial cells/neurons of neonatal SGNs increased over time, from 2.3 ± 0.6 (4 DIV) to 5.1 ± 1.2 (7 DIV) on CMOS chips and from 3.2 ± 0.9 (4 DIV) to 8.6 ± 3.7 (7 DIV) on control glass coverslips, due to glial cell proliferation. On the other hand, the opposite effect was found for adult neurons, since ratio of glial cells/neurons decreased over time, from 2.6 ± 1.0 to 2.1 ± 0.7 (4 DIV) on CMOS chips and from 3.6 ± 1.3 to 2.9 ± 1.1 (7 DIV) on control glass coverslips. This might be related to the age of animals and animal species, since other studies^13,14,49,50,54^ reported that the density of non-neuronal cells decreased with increasing age of the animal which can explain why dissociated SGN cultures of mature animals could be maintained for several weeks. The proliferation of glial cells was slower on micro-patterned surfaces of the CMOS chip in both, neonatal and adult SGN cultures, shown by lower ratios of glial cells/neurons, which increase the survival rate of neurons on these areas. Schwann and satellite cells are the most commonly SGN-associated cells in the spiral ganglion or spiral lamina, and they are immune-reactive for the marker S100^15,46^. These types of glial cells play an important role in neuronal growth, survival, regeneration and axonal guidance *in vivo* and *in vitro*^15,20,27,53^. It has been shown that SGNs in culture prefer to grow in proximity to glial cells, due to the adhesive molecular patterns like Laminin-1, that these cells secrete^15,20,27^. Our data show that neurites of the neonatal and adult SGNs on CMOS substrates were encouraged to grow more in direct contact with micro-pillars and micro-electrodes rather than to seek interaction with glial cells that were present in their surroundings. In fact, SGNs (Figs 2b and 3b) intertwined their neurites exchanging information from the environment instead being in the contact with glial cells. Neonatal SGNs have shown a tendency towards highly patterned neuronal networks promoting connections between neurites while discouraging contacts with glial cells. Previous studies by several groups^18,21,22,27,28^ demonstrated cytoskeletal alterations and upstream signaling related to neurons interacting with pillars and grooves indicating consistency with our findings. This suggests that topographic cues presented with micro-pillars on CMOS chips support direct neurite guidance without the mediating action of glial cells, creating an advantageous environment for neuronal growth and neurite sprouting. The mature mammalian cochlea exhibits segregated innervation of its two populations of sensory hair cells: inner hair cells (IHC) and outer hair cells (OHC), by the SGNs^39,51^. Type I SGNs comprise 90 to 95% of the SGN population and extend single, unbranched, myelinated neurites to innervate a single inner hair cell (IHC)^39,51^. The IHCs with Type I SGN innervation are responsible for the signal transduction of sound stimuli to be delivered up-stream along the auditory pathway^39,51^. The remaining 5 to 10% of SGN are Type II neurons with thin, unmyelinated fibers innervating numerous outer hair cells (OHCs)^39,51^. Adult SGNs cultured on glass coverslips and CMOS chips were stained with Tuj and peripherin to distinguish between Type I and Type II of SGNs. Peripherin positive (appeared in green)^39^ neurons were identified as Type II SGNs (Fig. 8). Only SGNs isolated from adult guinea pigs were stained with peripherin antibody because neonatal Type II SGNs occurred between 6 to 8 DIV and at that time-point it was impossible to distinguish between two neuronal types as observed morphological differences develop only later^40,42^. Consequently, rat pups are an inappropriate model to investigate the appearance of Type II SGNs. The presence of Type II SGNs on CMOS chips indicates the ability of the micro-structured surfaces to promote the natural appearances of both types of SGNs that are important for natural cochlear innervation. Spiral ganglion neurons have unusual morphological configuration in which electrogenic soma reside directly in the signal transduction pathway representing internodal axonal structure that serves for transmitting, integrating and conducting signals^48^. The position of the micro-electrodes within the CMOS chip is certainly an important aspect for electrophysiological recordings. The electrical activity of the SGNs can presumably be recorded if an electrogenic soma is positioned directly on the top of a micro-electrode or the neurite spans the micro-electrode ensuring neuron-electrode proximity. In this study, considering the extremely demanding and sensitive *in vitro* conditions for peripheral sensory neurons, we showed that there is a satisfactory number of SGNs either with soma placement directly on the top of a micro-electrode or a neurite spanning a micro-electrode, or both, which represent realistic requirements for electrophysiological applications. These findings were confirmed with live calcium imaging experiments of SGN cultures which were electrically stimulated with the CMOS electrode array. We observed that neurons with either soma positioned directly on the top of a micro-electrode or the neurite contacting the micro-electrode have shown a strong increase in the [Ca^2+^]_i_ after being stimulated with biphasic pulse trains. The fast increase in the [Ca^2+^]_i_ could be caused by two mechanisms: opening of voltage-gated Ca^2+^ channels causing influx of extracellular Ca^2+^ during stimulation and formation of pores along the membrane allowing extracellular Ca^2+^ to flow inside the cell. One of the first studies that utilize MEAs for assessing electrophysiological characteristics of SGNs *in vitro* was from Hahnewald *et al*.^9^. They used SGN explants and a MEA containing 68 platinum electrodes with a dimension of 40 × 40 µm and an inter-electrode distance of 200 µm and showed that the stimulation and recording of auditory neuronal activity from SGN explants using MEA is feasible. In our study we presented MEA with a much higher number of integrated microelectrodes that enables successful electrical stimulation of dissociated SGN cultures. We showed that the pillars of CMOS electrode array maintain a stable environment for SGNs cultured *in vitro*, while microelectrodes enable electrophysiological stimulation of each individual neuron. A smaller inter-electrode distance of the CMOS electrode array encourages a closer contact between auditory neurons and electrodes that can result in lower stimulation thresholds. Based on the position of the soma relative to the central process (usually referred to as the axon) and the peripheral process (usually referred to as the dendrite), neurite width asymmetry between these two processes in bipolar neurons can be observed in Type I SGN^39^. This asymmetry is shown in SEM images (Fig. 11) and was statistically quantified. Overall, these findings suggest micro-patterned and high-density complementary metal–oxide–semiconductor electrode array as a promising model of a neuro-electronic interface which supports neuronal growth, alignment, orientation of *in vitro* cultured spiral ganglion neurons and which has an ability to electrically stimulate neurons. We assume that electrical cues of micro-patterned and high-density CMOS electrode array would enable growth stimulation of regenerating neurites of spiral ganglion neurons attracting them toward electrodes. Future *in vitro* and *in vivo* studies will be necessary to elucidate the electrophysiological properties of auditory neurons interfaced with types of micro-patterned and high-density CMOS electrode arrays.
